# Effectiveness of an intervention to reduce sedentary behaviour as a personalised secondary prevention strategy for patients with coronary artery disease: main outcomes of the SIT LESS randomised clinical trial

**DOI:** 10.1186/s12966-023-01419-z

**Published:** 2023-02-14

**Authors:** B. M. A. van Bakel, S. H. Kroesen, E. A. Bakker, R. V. van Miltenburg, A. Günal, A. Scheepmaker, W. R. M. Aengevaeren, F. F. Willems, R. Wondergem, M. F. Pisters, M. de Bruin, M. T. E. Hopman, D. H. J. Thijssen, T. M. H. Eijsvogels

**Affiliations:** 1 Department of Physiology, Radboud Institute for Health Sciences, Radboud University Medical Center, P.O. Box 9101, 6500 HB Nijmegen, The Netherlands; 2 Department of Cardiology, Bernhoven Hospital, Uden, The Netherlands; 3 Department of Cardiology, Rijnstate Hospital, Arnhem, The Netherlands; 4 Department of Rehabilitation, Physical Therapy Science and Sport, University Medical Center Utrecht Brain Center, Utrecht University, Utrecht, The Netherlands; 5 Research Group Empowering Healthy Behaviour, Department of Health Innovations and Technology, Fontys University of Applied Sciences, Eindhoven, The Netherlands; 6 Center for Physical Therapy Research and Innovation in Primary Care, Julius Health Care Centers, Utrecht, the Netherlands; 7 Department of IQ Healthcare, Radboud Institute for Health Sciences, Radboud University Medical Center, Nijmegen, The Netherlands; 8 Research Institute for Sports and Exercise Sciences, Liverpool John Moores University, Liverpool, UK

**Keywords:** Cardiac rehabilitation, e-Health, Prevention, Sedentary lifestyle, Physical activity, Cardiovascular disease

## Abstract

**Background:**

A high sedentary time is associated with increased mortality risk. Previous studies indicate that replacement of sedentary time with light- and moderate-to-vigorous physical activity attenuates the risk for adverse outcomes and improves cardiovascular risk factors. Patients with cardiovascular disease are more sedentary compared to the general population, while daily time spent sedentary remains high following contemporary cardiac rehabilitation programmes. This clinical trial investigated the effectiveness of a sedentary behaviour intervention as a personalised secondary prevention strategy (SIT LESS) on changes in sedentary time among patients with coronary artery disease participating in cardiac rehabilitation.

**Methods:**

Patients were randomised to usual care (*n* = 104) or SIT LESS (*n* = 108). Both groups received a comprehensive 12-week centre-based cardiac rehabilitation programme with face-to-face consultations and supervised exercise sessions, whereas SIT LESS participants additionally received a 12-week, nurse-delivered, hybrid behaviour change intervention in combination with a pocket-worn activity tracker connected to a smartphone application to continuously monitor sedentary time. Primary outcome was the change in device-based sedentary time between pre- to post-rehabilitation. Changes in sedentary time characteristics (prevalence of prolonged sedentary bouts and proportion of patients with sedentary time ≥ 9.5 h/day); time spent in light-intensity and moderate-to-vigorous physical activity; step count; quality of life; competencies for self-management; and cardiovascular risk score were assessed as secondary outcomes.

**Results:**

Patients (77% male) were 63 ± 10 years and primarily diagnosed with myocardial infarction (78%). Sedentary time decreased in SIT LESS (− 1.6 [− 2.1 to − 1.1] hours/day) and controls (− 1.2 [ ─1.7 to − 0.8]), but between group differences did not reach statistical significance (─0.4 [─1.0 to 0.3]) hours/day). The post-rehabilitation proportion of patients with a sedentary time above the upper limit of normal (≥ 9.5 h/day) was significantly lower in SIT LESS *versus* controls (48% *versus* 72%, baseline-adjusted odds-ratio 0.4 (0.2–0.8)). No differences were observed in the other predefined secondary outcomes.

**Conclusions:**

Among patients with coronary artery disease participating in cardiac rehabilitation, SIT LESS did not induce significantly greater reductions in sedentary time compared to controls, but delivery was feasible and a reduced odds of a sedentary time ≥ 9.5 h/day was observed.

**Trial registration:**

Netherlands Trial Register: NL9263.

**Graphical Abstract:**

Outcomes of the SIT LESS trial: changes in device-based sedentary time from pre-to post-cardiac rehabilitation (control group) and cardiac rehabilitation + SIT LESS (intervention group). SIT LESS reduced the odds of patients having a sedentary time >9.5 hours/day (upper limit of normal), although the absolute decrease in sedentary time did not significantly differ from controls. SIT LESS appears to be feasible, acceptable and potentially beneficial, but a larger cluster randomised trial is warranted to provide a more accurate estimate of its effects on sedentary time and clinical outcomes. CR: cardiac rehabilitation.

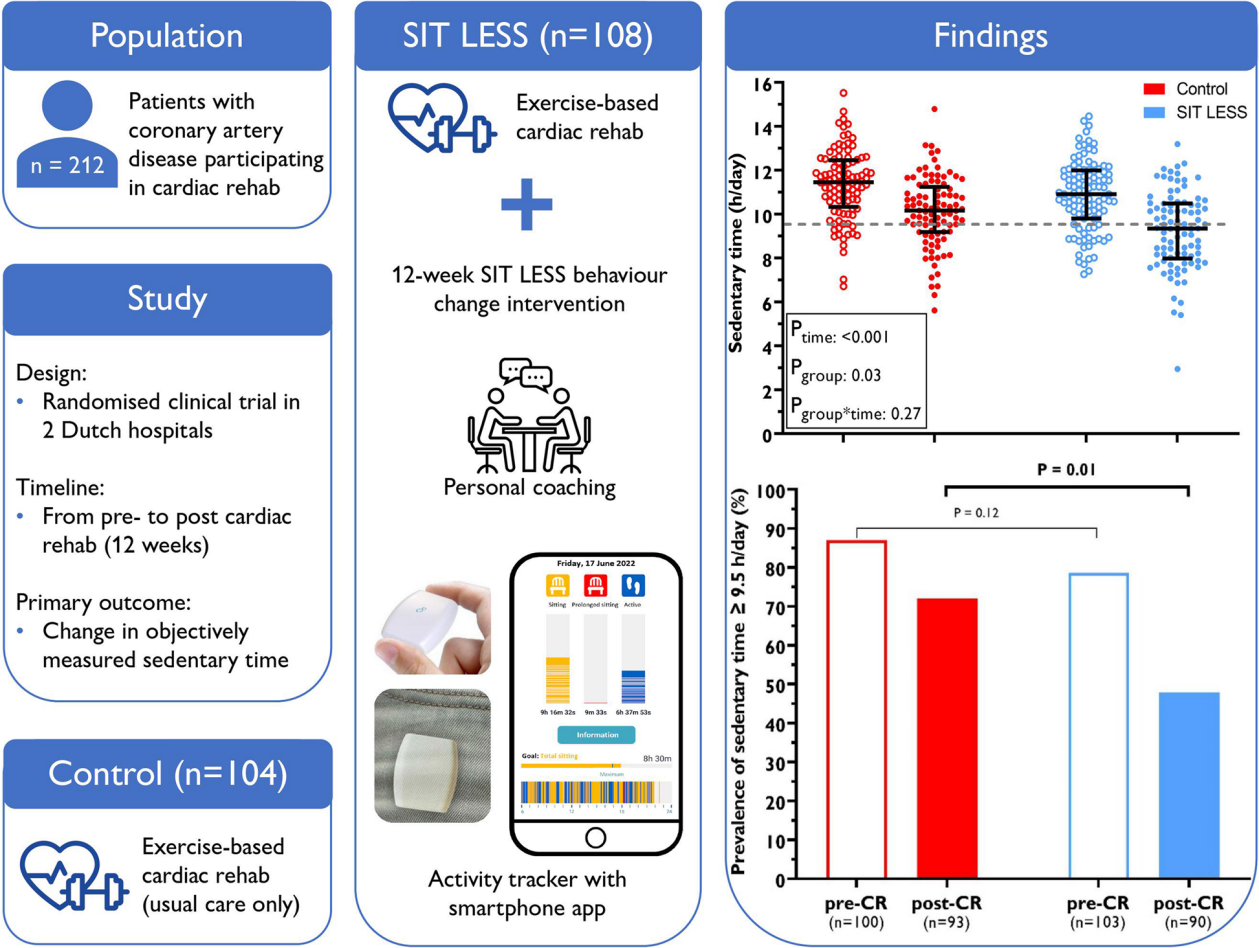

**Supplementary Information:**

The online version contains supplementary material available at 10.1186/s12966-023-01419-z.

## Background

Sedentary behaviour is defined as any low-intensity behaviour (energy expenditure ≤ 1.5 Metabolic Equivalent of Task) while awake in a sitting, lying or reclining posture [1]. Emerging evidence indicates that a daily sedentary time (ST) exceeding the upper limit of normal (i.e. 9.5 h/day) is associated with an increased risk of cardiovascular disease morbidity and mortality, even after accounting for traditional risk factors [2–4]. A sedentary lifestyle is highly prevalent among patients with coronary artery disease (CAD), as evidenced by substantially higher ST compared to the general population (10.4 *versus* 9.4 h/day) [5, 6].

Epidemiological studies have shown that replacement of ST with light and moderate-to-vigorous physical activity attenuates the risk for adverse outcomes in cardiovascular disease patients and the general population [5, 7, 8]. Furthermore, interruption of ST by taking active breaks can also improve cardiovascular risk factors and health outcomes [9, 10]. Exercise-based cardiac rehabilitation (CR) aims to enhance habitual physical activity of patients with CAD [11], but programmes typically do not target ST. Consequently, reductions in ST following CR are only small (0.2 – 0.4 h/day) [6, 12, 13], and absolute ST remains high among CR graduates [14, 15]. Interventions that specifically target ST in patients with CAD are currently lacking. Therefore, effective strategies to reduce ST are paramount to improve secondary prevention in patients with CAD.

Behaviour change interventions are recommended to be based on a theoretical framework, tailored to the target population and should consist of multiple behaviour change strategies to create a maximal effect among male participants [16]. For example, monitoring the targeted behaviour in combination with motivational interviewing with goal-setting and coping planning has been shown to be a valuable behaviour change method [17–19]. To efficiently implement these elements in real-world clinical settings, a centre-based CR programme can be extended with home-based digital health solutions and telephone coaching sessions, after addressing concerns about digital literacy, data safety and privacy [20]. Nevertheless, the COVID-19 pandemic has paved the way for large-scale implementation of digital health and wearable monitoring devices in hybrid (centre- and home-based) CR programmes [21]. In close collaboration with patients and healthcare providers we used an intervention mapping adaptation framework [17] to adapt a previously developed, successful and cost-effective self-management intervention addressing multiple behavioural determinants (knowledge, motivation and self-regulation) in clinical settings [22]. We combined it with the use of an activity tracker with real-time vibrotactile feedback that successfully reduced ST in obese patients [19] and tailored it to the needs of patients with CAD, as previously described in detail elsewhere [23]. To the best of our knowledge, effectiveness of a multicomponent, hybrid behaviour change intervention with a primary focus on reducing and interrupting sedentary time in patients with CAD during CR has not been studied before.

Our primary objective was to evaluate the effectiveness of a Sedentary Behaviour Intervention as a Personalised Secondary Prevention Strategy (SIT LESS) on changes in device-based ST among patients with CAD participating in CR. Changes in ST characteristics (prevalence of prolonged sedentary bouts ≥ 30 min and proportion of patients with ST ≥ 9.5 h/day); time spent in light-intensity and moderate-to-vigorous physical activity; step count; quality of life (Heart-QoL); competencies for self-management (PAM-13); and cardiovascular risk score (SMART-score) were assessed as secondary outcomes. Additionally, we performed subgroup analyses based on patient- and disease characteristics to identify whether the change in ST was similar across predefined subgroups of patients. Other outcome measures focused on process evaluation. For this purpose, the number of CR consultations and exercise sessions attended was assessed. We also determined the number of valid wear days of the pocket-worn activity tracker, telephone consultations and the course of ST throughout the intervention period. In this randomised clinical trial, we hypothesised that adding SIT LESS to CR would reduce ST to a greater extent compared to controls.

## Methods

### Setting and population

A randomised clinical trial was conducted in two Dutch hospitals in order to determine the effectiveness of SIT LESS: a 12-week, hybrid and personalised behaviour change intervention in addition to CR, to reduce ST in patients with CAD (Netherlands Trial Register: NL9263). The rationale and design of the SIT LESS trial has previously been described in detail [23]. Patients from Bernhoven Hospital (Uden, The Netherlands) and Rijnstate Hospital (Arnhem, The Netherlands) were included in this trial. Patients aged ≥ 18 years old were eligible for participation if referred to CR because of stable CAD, an acute coronary syndrome, and/or after coronary revascularisation. Furthermore, they had to be able to understand and perform study related procedures such as sufficient digital knowledge to use smartphone applications. Exclusion criteria were heart failure (New York Heart Association (NYHA) class III or IV); physically unable to stand or walk (e.g. wheelchair-bounded); an expected coronary artery bypass grafting (CABG) within eight weeks after inclusion; and coincident participation in another interventional trial targeting ST or physical activity [23]. The SIT LESS trial was approved by the Medical Ethics Committee of the Radboud university medical center (#2020–6101), and all participants gave written informed consent.

### Randomisation

Participants were randomly allocated (1:1) into the control- or SIT LESS group in random block sizes ranging from four to six, using a computerised algorithm (Castor Electronic Data Capture 2021, Ciwit B.V., Amsterdam, The Netherlands). Randomisation was stratified by sex and hospital to ensure balance of the treatment arms. All participants who withdrew within the first two weeks after inclusion were replaced to ensure sufficient power to assess our primary outcome. Due to the nature of the intervention, nurse specialists and patients were not blinded to the treatment allocation.

### Usual care

All participants in the SIT LESS trial received usual care, consisting of a comprehensive CR programme, delivered by healthcare professionals such as nurse specialists and physical therapists, with a total duration of ~ 12 weeks (usual care). One to three regular, individual consultations were scheduled with the nurse specialist focusing on lifestyle, medication and psychosocial wellbeing. Participants were offered an outpatient physical activity programme, consisting of ~ 12 supervised, one hour exercise group sessions across six weeks.

### SIT LESS intervention

**Fig. 1:**
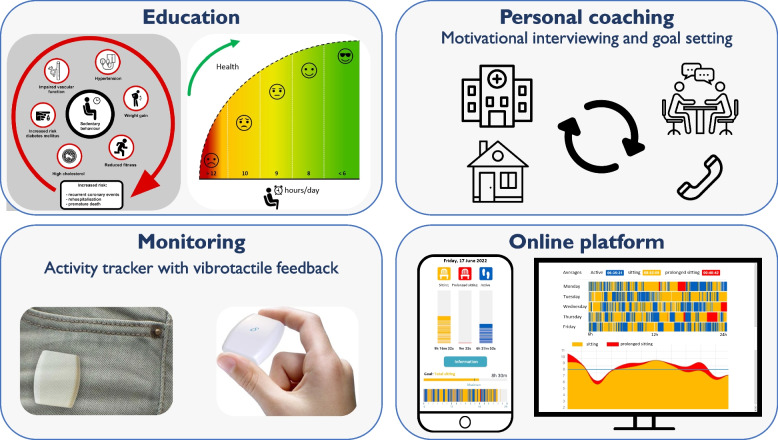
Impression of the multicomponent SIT LESS intervention, a 12-week, personalised, nurse-delivered and hybrid programme consisting of 1) patient education regarding sedentary behaviour; 2) personal coaching using motivational interviewing techniques during face-to-face consultations in the hospital and telephone consultations at home; 3) monitoring of time spent sedentary using a pocket-worn activity tracker providing vibrotactile feedback after a predefined limit for sedentary bouts was exceeded; and 4) online platform with smartphone application (connected to the activity tracker) and web-based dashboard to enable 24/7 feedback and (remote) coaching

Patients in the SIT LESS group received the SIT LESS intervention alongside usual care CR. SIT LESS was developed in close collaboration with patients and nurse specialists following the intervention mapping adaptation framework [17]. According to these principles, we adapted an already existing, successful self-management intervention for clinical settings [22] and added the use of a pocket-worn activity tracker (Activ8sit, 2 M Engineering, Valkenswaard, The Netherlands) that previously successfully reduced ST in obese individuals [19]. SIT LESS was also tailored to the needs of patients with CAD by evaluating the adapted intervention in two advisory board meetings. The advisory board consisted of three patients with CAD and was consulted twice during the development of SIT LESS. After processing feedback of the first meeting, the advisory board critically appraised the full manual and design of the SIT LESS intervention again, ultimately reaching unanimous consensus about the content. This resulted in a 12-week, personalised, nurse-delivered and hybrid behaviour change intervention. The multicomponent SIT LESS programme consists of 1) patient education; 2) goal-setting; 3) motivational interviewing with coping planning; and 4) (tele)monitoring using a pocket-worn activity tracker connected to a smartphone application (RISE, Appbakkers B.V., Zwolle, The Netherlands) and providing vibrotactile feedback after a predefined limit for sedentary bouts (e.g. 30 min) was exceeded (Fig. 1). The predefined limit of prolonged sedentary bouts was by default set at ≥ 30 min, based on studies showing that sedentary bouts > 30 min are associated with a higher risk of all-cause mortality compared to sitting time accumulated in shorter sedentary bouts (1 to 29 min) [24, 25]. The SIT LESS programme was personalised at individual level, for example regarding the plan to achieve the selected goal of daily ST with discussing difficult situations and possible solutions that are applicable to the individual patient in the outpatient clinic.

Patients received three face-to-face consultations for SIT LESS coaching provided by the nurse specialists. During these consultations the SIT LESS manual (Supplemental Document 1) was used in which each step of the intervention was described in detail, ultimately covering all core components of SIT LESS. The initial steps focused on patient education to enhance patient’s knowledge regarding the risk of sedentary behaviour and benefits of reducing ST using visual materials. Subsequently motivation to reduce ST was discussed, focusing on underlying personal reasons and addressing possible concerns. In the next step, patients set their goal of maximal daily ST in combination with an expected scenario regarding their pattern of sedentary behaviour during the upcoming period until the next consultation. Based on the selected scenario, patients were challenged to explain personal reasons for sitting less and to identify potential barriers. Motivational interviewing is an important part of patient counselling, and adopts language designed to strengthen personal motivation and commitment to a specific goal [17]. To improve chances to achieve the planned outcomes, a specific plan (IF–THEN planning) was defined and challenging situations and possible solutions were discussed. In the next step, the patient’s confidence achieving the goal was determined and reasons for change were reinforced, while potential residual barriers were evaluated and discussed. Additionally, the pocket-worn activity tracker connected to a smartphone application enabled patients and nurse specialists to register and adjust personal goals; and upload and review daily ST. This is a commercially available, small (30 × 32 × 10 mm), lightweight (20 g) and validated device to monitor ST [26]. Based on the activity tracker-derived ST reports, patients were contacted by telephone for supportive coaching throughout the intervention period. During week 1 to 6, telephone coaching took place on a weekly basis, followed by bi-weekly telephone coaching during week 7 to 12. The participating nurse specialists were registered with the Dutch Association for Cardiovascular Nurses and were trained in the basics of motivational interviewing as part of their education. Prior to delivering SIT LESS to the intervention group, all nurse specialists received a comprehensive and accredited training course under the guidance of a behavioural psychologist. The training course consisted of a self-study document (± 3 h of preparation), followed by an on-site group training (± 8 h) where theory on motivational interviewing techniques was explained and subsequently practiced through active role-play. The development, content and timeline of SIT LESS has been reported in detail elsewhere [23].

### Outcomes

The primary outcome was the change in device-based ST, expressed in hours per day, from pre-CR to post-CR. Changes in ST characteristics (i.e. prevalence of prolonged sedentary bouts (≥ 30 min) and proportion of patients with ST ≥ 9.5 h/day); light-intensity physical activity (LIPA); moderate-to-vigorous intensity physical activity (MVPA); step count; quality of life (HeartQoL [27]); patients’ competencies for self-management (PAM-13 [28]) and 10-year risk of recurrent cardiovascular events (SMART risk score [29]) were assessed as secondary outcomes. Other outcome measures included process evaluation (i.e. activity tracker data (adherence, week-to-week sedentary time and number of prolonged sedentary bouts); evaluation of study participation (10-point scale), number of CR consultations attended; and number of supervised exercise sessions).

### Measurements

ST and physical activity were objectively assessed using a validated accelerometer (ActivPAL3^TM^micro, PAL Technologies Ltd., Glasgow, United Kingdom) [30]. The ActivPAL is a small device (25 × 45x5 mm), attached to the patient’s thigh using hypoallergenic tape and sealed with a nitrile sleeve and transparent tape for waterproof protection. The ActivPAL combines a tri-axial accelerometer with an inclinometer which accurately distinguishes between sitting, standing and walking [30]. Patients were instructed to wear the ActivPAL 24 h/day for 8 consecutive days and to fill in a sleep diary pre- and post-CR. After the ActivPAL was returned to our research institute by mail, raw data were analysed by a modified version of the script of Winkler et al. [31]. Total ST was expressed in hours per day and accumulation of ST was examined by calculating the number of prolonged (≥ 30 min) sedentary bouts. The daily ST was dichotomised indicating whether each participant was above or below the upper limit of normal (i.e. 9.5 h/day) at pre- and post-CR. Physical activities were categorised as LIPA (Metabolic Equivalent of Task < 3) or MVPA (Metabolic Equivalent of Task ≥ 3) and expressed in hours per day, whereas step count was expressed as number of steps per day.

For patients randomised to the SIT LESS arm, we calculated the number of valid wear days of the activity tracker (≥ 10 h/day) to assess the adherence by dividing the number of valid wear days by the total number of days of the intervention period. Additionally, we evaluated the course of ST throughout the intervention period (i.e. weekly average of ST (hours/day) and the number of prolonged sedentary bouts).

Patient characteristics, CR characteristics (i.e. number of CR consultations attended; and number of supervised exercise sessions) and clinical predictors relating to assessment of the SMART risk score were derived from the electronic patient files. After inclusion, the HeartQoL and PAM-13 questionnaires were completed, and socio-economic status and ethnicity was assessed. After 12 weeks, patients received an online questionnaire to re-evaluate the HeartQoL and PAM-13 at post-CR.

### Statistical analysis

We estimated that a sample size of 212 participants (106 per arm) would ensure 80% power to detect a 0.5 h/day ST difference between the two arms, using a two-sided type I error rate of 0.05 and assuming a standard deviation of 1.18 h/day. The calculation was based on prior studies and assumed a 15% dropout rate [6, 13, 32].

Primary outcome analysis was performed on an intention-to-treat basis. Between-group difference in the change in ST from pre- to post-CR was evaluated using linear mixed model analysis using random intercepts with time (pre- to post-CR) as categorical variable. Subgroup analyses were performed based on patient characteristics, including sex, age, employment status, living environment and education level as well as disease characteristics, including index diagnosis and treatment. We evaluated the difference between the control- and SIT LESS group in the proportion of patients with ST ≥ 9.5 h/day at post-CR by logistic regression analysis and adjustment for baseline ST. For other secondary outcomes, we used mixed model analysis to assess changes in the number of prolonged sedentary bouts per day; time spent in LIPA and MVPA per day; daily step count; HeartQoL, PAM score and SMART risk score from pre- to post-CR. Within the SIT LESS group, week-to-week differences in ST and prolonged sedentary bouts were investigated using mixed model analyses using random intercepts with time as continuous variable.

Normally distributed continuous variables were presented as mean ± standard deviation and non-normally variables as median [interquartile range]. Categorical variables were expressed as numerical values and percentages. All statistical tests were two-sided, confidence intervals were at the 95% level and *P*-values < 0.05 were considered statistically significant. Analyses were performed using SPSS statistics 25.0 (IBM, Chicago, USA).

## Results

### Patient characteristics

**Fig. 2:**
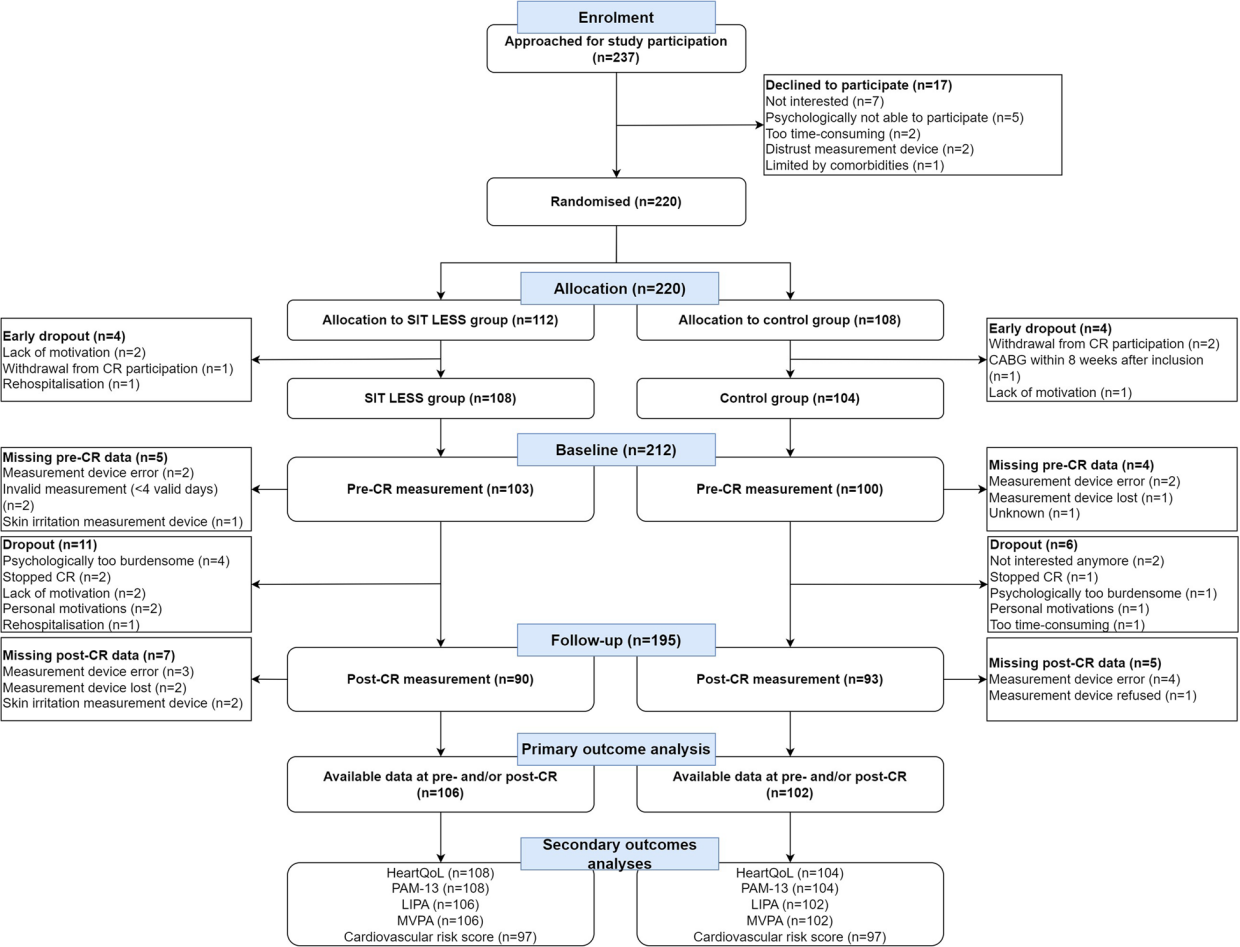
CONSORT flowchart of the SIT LESS randomised clinical trial. In total 237 patients were approached for participation, of which 220 were randomised to either to SIT LESS group or the control group. Eight patients dropped out prior to CR initiation, leaving 108 patients in the SIT LESS group and 104 in the control group. In the SIT LESS group, collected data at pre- and/or post CR was available for primary analysis in 106 patients *versus* 102 patients in the control group

Between 30 March and 23 December 2021, 237 patients were approached for study participation, of which 220 were randomly assigned to the control group or SIT LESS group. Eight patients dropped out within two weeks after randomisation (Fig. 2). Recruitment was equally distributed between both hospitals (Bernhoven hospital: *n* = 107; Rijnstate hospital: *n* = 105) and patient characteristics were well balanced between the two treatment arms (Table 1). The age of participants was 63 ± 10 years, 164 (77%) were male, and index diagnosis was primarily non-ST-elevated myocardial infarction (NSTEMI, 48%) or ST-elevated myocardial infarction (STEMI, 30%). CR completion rate (88%), including supervised exercise sessions did not differ between groups (*p* = 0.98) (Supplemental Table 1). During the study period, there were 6 (6%) dropouts in the control group and 11 (10%) in the SIT LESS group (Fig. 2). 97 out of 108 (90%) patients in the SIT LESS group completed all face-to-face SIT LESS coaching consultations during an intervention period of 89 ± 13 days. During this period, median number of telephone coaching sessions was 7 [6-8] and the adherence to the use of the activity tracker across the entire intervention period was 84 [72–94]% (Supplemental Table 2). Reasons for premature discontinuation of the activity tracker (11%) were summarised in Supplemental Table 3.

**Table 1:** Patient and disease-related characteristics of the study cohort

	**Total population (*n* = 212)**	**Missing values (n (%))**	**SIT LESS group (*n* = 108)**	**Control group (*n* = 104)**
**Patient characteristics**				
**Age (years)**	63 (± 10)	0 (0%)	63 (± 10)	64 (± 10)
**Sex (female)**	48 (23%)	0 (0%)	25 (23%)	23 (22%)
**Body mass index (kg/m**^**2**^**)**	27.1 [24.6–30.2]	0 (0%)	27.1 [24.6–30.1]	27.2 [24.6–30.8]
**Ethnicity**		0 (0%)		
Dutch (n (%))	201 (95%)		104 (96%)	97 (93%)
Asian (n (%))	4 (2%)		0 (0%)	4 (4%)
European other (n (%))	3 (2%)		1 (1%)	2 (2%)
South-American (n (%))	3 (1%)		2 (2%)	1 (1%)
African (n (%))	1 (0%)		1 (1%)	0 (0%)
**Education level**		20 (9%)		
Low (n (%))	46 (24%)		19 (20%)	27 (28%)
Middle (n (%))	77 (40%)		38 (40%)	39 (30%)
High (n (%))	69 (36%)		38 (40%)	31 (32%)
**Living together/married**	152 (79%)	20 (9%)	77 (81%)	75 (77%)
**Working status**				
**Employed**	98 (51%)	20 (9%)	53 (49%)	45 (46%)
Mainly sedentary at work (n (%))	35 (36%)		20 (38%)	15 (33%)
Some light physical activities at work (n (%))	20 (20%)		13 (24%)	7 (16%)
Light to moderate physical activities at work (n (%))	10 (10%)		4 (8%)	6 (13%)
Moderate to vigorous physical activities at work (n (%))	33 (34%)		16 (30%)	17 (38%)
**Unemployed**	94 (44%)	20 (9%)	42 (44%)	52 (54%)
Retirement (n (%))	80 (85%)		37 (88%)	43 (83%)
Health problems (n (%))	13 (14%)		4 (10%)	9 (17%)
Household tasks (n (%))	1 (1%)		1 (2%)	0 (0%)
**Monthly household income (€)**		37 (17%)		
< 2000 (n (%))	35 (20%)		19 (22%)	16 (17%)
2000 – 3999 (n (%))	95 (54%)		44 (52%)	51 (56%)
4000 – 5999 (n (%))	35 (20%)		18 (21%)	17 (19%)
≥ 6000 (n (%))	10 (6%)		4 (5%)	6 (8%)
**Living environment**		19 (9%)		
Transition (n (%))	89 (46%)		43 (45%)	46 (47%)
Urban (n (%))	54 (28%)		23 (24%)	31 (32%)
Rural (n (%))	50 (26%)		29 (31%)	21 (21%)
**Regular step count tracking by smartwatch or smartphone (n (%))**	71 (34%)	0 (0%)	33 (31%)	38 (37%)
**Lifestyle factors**				
**Alcohol use (n (%))**	168 (79%)	0 (0%)	87 (81%)	81 (78%)
Current drinker (n (%))	146 (87%)		75 (86%)	71 (88%)
Units/week (n)	5 [2-10]		5 [3-9]	5 [2-14]
**Smoking (n (%))**	150 (71%)	0 (0%)	73 (68%)	77 (74%)
Current smoker (n (%))	41 (27%)		19 (26%)	22 (28%)
Packyears (n)	23 [10-36]		23 [10-37]	22 [10-36]
**Medical history**				
**Comorbidities**		0 (0%)		
Hypertension (n (%))	85 (40%)		41 (38%)	44 (42%)
Dyslipidaemia (n (%))	66 (31%)		34 (32%)	32 (31%)
Diabetes mellitus (n (%))	36 (17%)		17 (16%)	19 (18%)
Prior myocardial infarction (n (%))	33 (16%)		18 (17%)	15 (14%)
Prior PCI (n (%))	26 (12%)		17 (16%)	9 (9%)
Prior CABG (n (%))	9 (4%)		7 (6%)	2 (2%)
Atrial fibrillation (n (%))	18 (9%)		7 (7%)	11 (11%)
Heart failure with reduced ejection fraction (n (%))	17 (8%)		10 (9%)	7 (7%)
Peripheral artery disease (n (%))	17 (8%)		8 (7%)	9 (9%)
Heart valve disease (n (%))	15 (7%)		12 (11%)	3 (3%)
Depression (n (%))	12 (6%)		7 (7%)	5 (5%)
Cancer (diagnosed in the past 5 years) (n (%))	12 (6%)		8 (7%)	4 (4%)
Rheumatoid arthritis (n (%))	8 (4%)		4 (4%)	4 (4%)
COPD (n (%))	8 (4%)		3 (3%)	5 (5%)
CVA (n (%))	8 (4%)		3 (3%)	5 (5%)
TIA (n (%))	7 (3%)		3 (3%)	4 (4%)
Chronic renal failure (eGFR < 30 ml/min/1.73m^2^ or dialysis) (n (%))	7 (3%)		6 (6%)	1 (1%)
**Hospitalisation**				
**Out of hospital cardiac arrest (n (%))**	7 (3%)	0 (0%)	4 (4%)	3 (3%)
**Index diagnosis**		0 (0%)		
Non-ST-elevation myocardial infarction (n (%))	102 (48%)		57 (53%)	45 (43%)
ST-elevation myocardial infarction (n (%))	64 (30%)		30 (28%)	34 (33%)
Stable angina pectoris (n (%))	30 (14%)		14 (13%)	16 (15%)
Unstable angina pectoris (n (%))	16 (8%)		7 (7%)	9 (9%)
**Coronary angiography findings**		0 (0%)		
1-vessel disease (n (%))	113 (53%)		60 (56%)	53 (51%)
2-vessel disease (n (%))	49 (23%)		22 (20%)	27 (26%)
3-vessel disease (n (%))	41 (19%)		20 (19%)	21 (20%)
No significant stenosis (n (%))	9 (4%)		6 (6%)	3 (3%)
**Treatment**		0 (0%)		
PCI (n (%))	126 (59%)		62 (57%)	64 (62%)
CABG (n (%))	56 (26%)		28 (26%)	28 (27%)
Conservative (optimal medical treatment only) (n (%))	30 (14%)		18 (17%)	12 (12%)
**Laboratory values**				
Peak hs-cTnT (ng/L) (Bernhoven cohort)	1977 [380–19151]	22 (21%)	2083 [437–24275]	1496 [352–16206]
Peak hs-cTnI (ng/L) (Rijnstate cohort)	5155 [500–23984]	10 (10%)	3668 [291–25000]	5554 [655–22276]
Peak CK (U/L)	199 [104–508]	34 (16%)	236 [113–526]	180 [99–475]
Total cholesterol (mmol/L)	5.0 (± 1.4)	36 (17%)	4.9 (± 1.2)	5.1 (± 1.5)
LDL-cholesterol (mmol/L)	3.0 (± 1.2)	37 (18%)	2.9 (± 1.1)	3.1 (± 1.3)
HDL-cholesterol (mmol/L)	1.1 (± 0.3)	36 (17%)	1.2 (± 0.4)	1.1 (± 0.3)
Triglycerides (mmol/L)	1.4 [1.0–2.2]	36 (17%)	1.4 [1.0–2.0]	1.5 [1.0–2.5]
**In-hospital complications (n (%))**	17 (8%)	0 (0%)	8 (8%)	9 (9%)
Complicated PCI (n (%))	6 (3%)		3 (3%)	3 (3%)
Complicated CABG (n (%))	5 (2%)		2 (2%)	3 (3%)
Target vessel revascularisation (n (%))	2 (1%)		1 (1%)	1 (1%)
Ischemic CVA (n (%))	2 (1%)		1 (1%)	1 (1%)
Major bleeding (n (%))	1 (0.5%)		0 (0%)	1 (1%)
In hospital cardiac arrest (n (%))	1 (0.5%)		1 (1%)	0 (0%)
**Duration of hospitalisation (days)**	5 [3-9]	0 (0%)	5 [3-9]	5 [3-10]

Data are presented as n (%) for categorical variables and as mean (± standard deviation) or median [interquartile range] for continuous variables

*CABG* Coronary artery bypass grafting, *COPD* Chronic obstructive pulmonary disease, *CK* Creatine kinase, *CVA* Cerebrovascular accident, *HDL* High-density lipoprotein, *hs-cTnI* High-sensitive cardiac Troponin-I, *hs-cTnT* High-sensitive cardiac Troponin-T, *LAD* Left anterior descending artery, *LDL* Low-density lipoprotein, *LM* Left main, *PCI* Percutaneous Coronary Intervention, *RCA* Right coronary artery, *RCx* Ramus circumflex artery, *TIA* Transient ischemic attack

### Sedentary time reduction

**Fig. 3:**
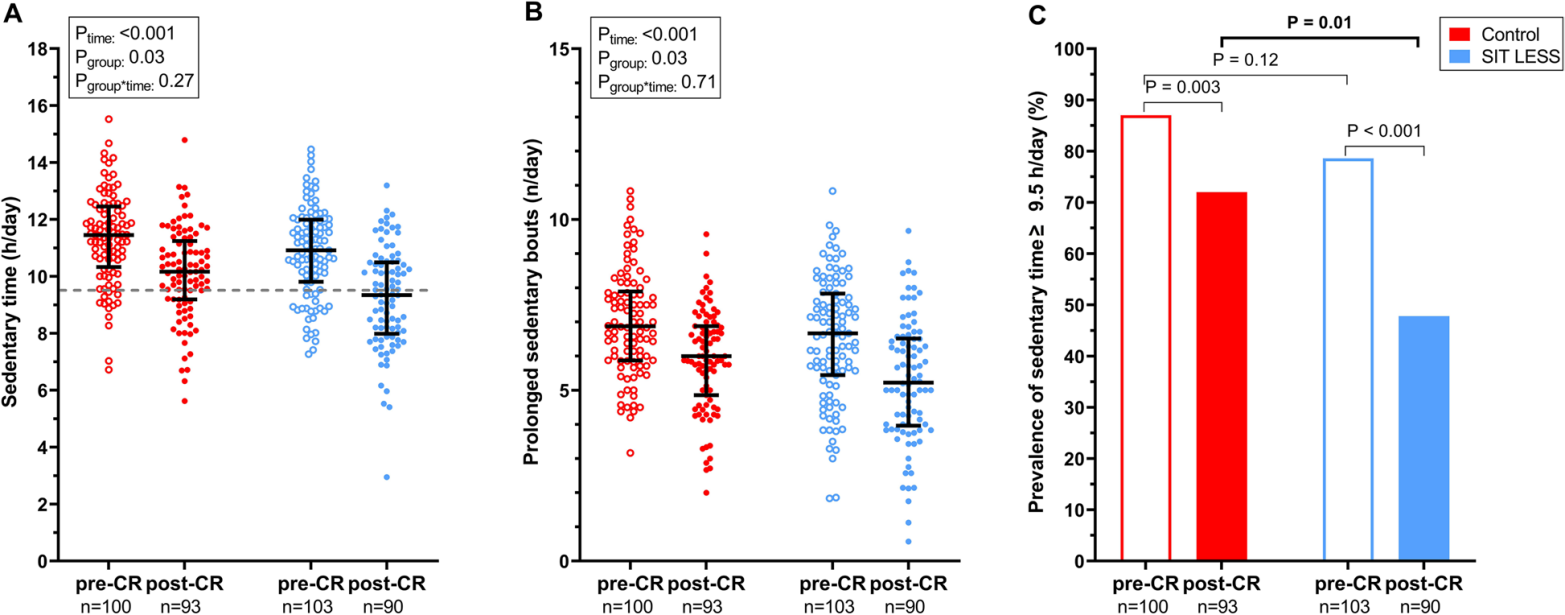
Sedentary behaviour outcomes of the SIT LESS randomised clinical trial in patients with coronary artery disease pre- and post-cardiac rehabilitation (CR). Panel **A** scatter plot of sedentary time with median and interquartile range compared between the control- (in red) and the SIT LESS group (in blue). The dashed line represents the upper-limit of normal daily sedentary time (9.5 h per day). *P*-values are based on mixed model analysis. Panel **B** scatter plot with of prolonged sedentary bouts (≥ 30 min per day) with median and interquartile range compared between the control- (in red) and the SIT LESS group (in blue). *P*-values are based on mixed model analysis. Panel **C** prevalence of sedentary time above the upper-limit of normal pre- and post-CR, with a significantly lower proportion of patients with a daily sedentary time above the upper-limit after CR in the SIT LESS group (in blue) compared to the control group (in red). The *p*-value representing the between group difference post-CR (*p* = 0.01) was adjusted for pre-CR sedentary time

For our primary outcome analysis, 106 (98%) patients in the SIT LESS group and 102 (98%) patients in the control group were available (Fig. 2). At pre-CR, daily ST was 11.3 ± 1.6 h/day in the control group and 10.9 ± 1.6 h/day in the SIT LESS group. Following CR, the change in ST was − 1.2 (95% confidence interval (CI) ─1.7; − 0.8)) hours/day in controls and − 1.6 (95% CI − 2.1; − 1.1) hours/day in SIT LESS. The difference in ST reduction between controls and SIT LESS did not reach statistical significance (─0.4 (95% CI ─1.0; 0.3) hours/day, *p* = 0.27) (Fig. 3, panel A). The effectiveness of SIT LESS to reduce ST was also not significantly different across pre-defined subgroups (Supplemental Fig. 1).

### Changes in sedentary time characteristics

The proportion of patients with ST ≥ 9.5 h/day was comparable between groups upon enrolment (79% *versus* 87%), but was significantly lower in the SIT LESS *versus* control group (48% *versus* 72%, *p* = 0.01) at post-CR (baseline-adjusted odds ratio 0.4 (95% CI 0.2–0.8)) (Fig. 3, panel C). The number of daily prolonged sedentary bouts reduced from pre- to post-CR, but the magnitude of this change did not differ between controls and SIT LESS (─0.1 (95% CI ─0.8; 0.5 bouts/day) (Fig. 3, panel B). Based on the pocket-worn activity-tracker data of the SIT LESS group, ST gradually declined from 8.9 [7.6–9.7] hours/day during the first week of the intervention period, to 7.8 [6.6–9.1] hours/day in the last week (estimate of weekly change in ST during intervention period: − 0.05 (95% CI ─0.08; − 0.01) hours/day, *p* = 0.01) (Supplemental Fig. 2).

### Physical activity, quality of life and cardiovascular risk score

**Fig. 4:**
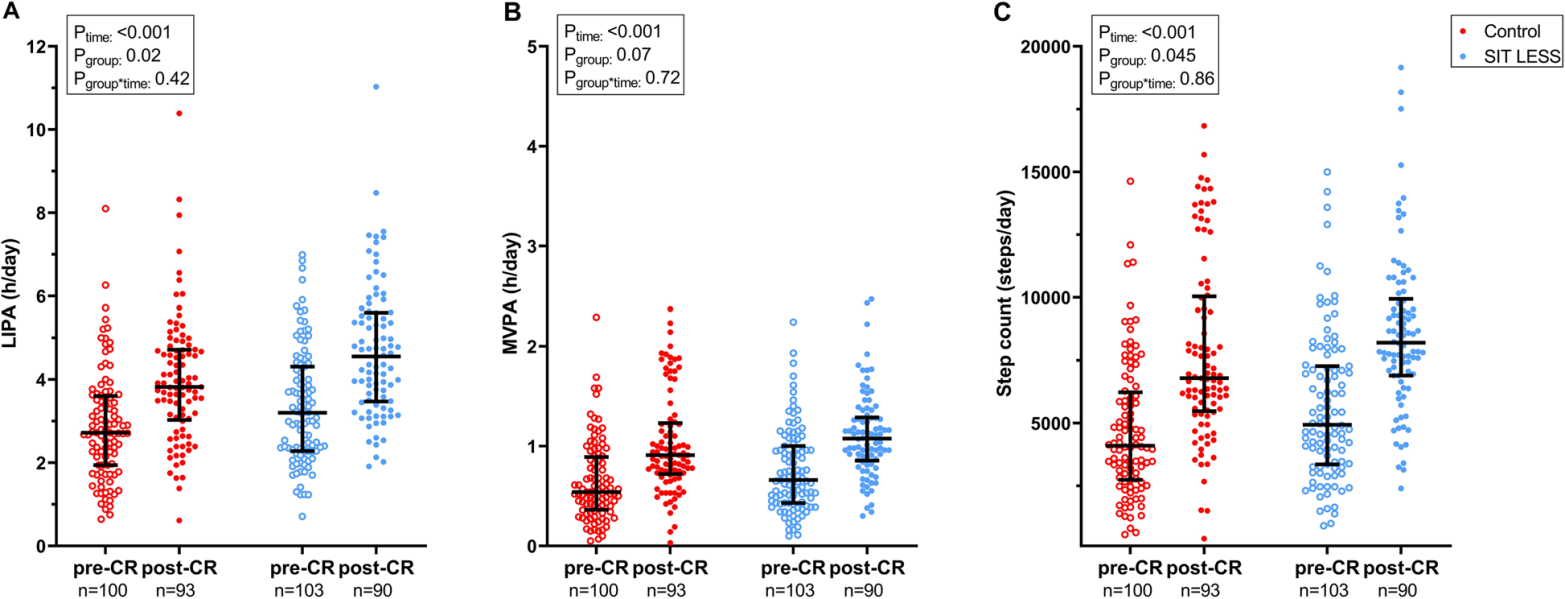
Physical activity outcomes of the SIT LESS randomised clinical trial in patients with coronary artery disease pre- and post-cardiac rehabilitation (CR). Scatter plots with median and interquartile range of light-intensity physical activity (LIPA, panel** A**); moderate-to-vigorous intensity physical activity (MVPA, panel **B**); and step count (panel **C**) compared between the control- (in red) and the SIT LESS group (in blue). *P*-values are based on mixed model analysis

**Fig. 5:**
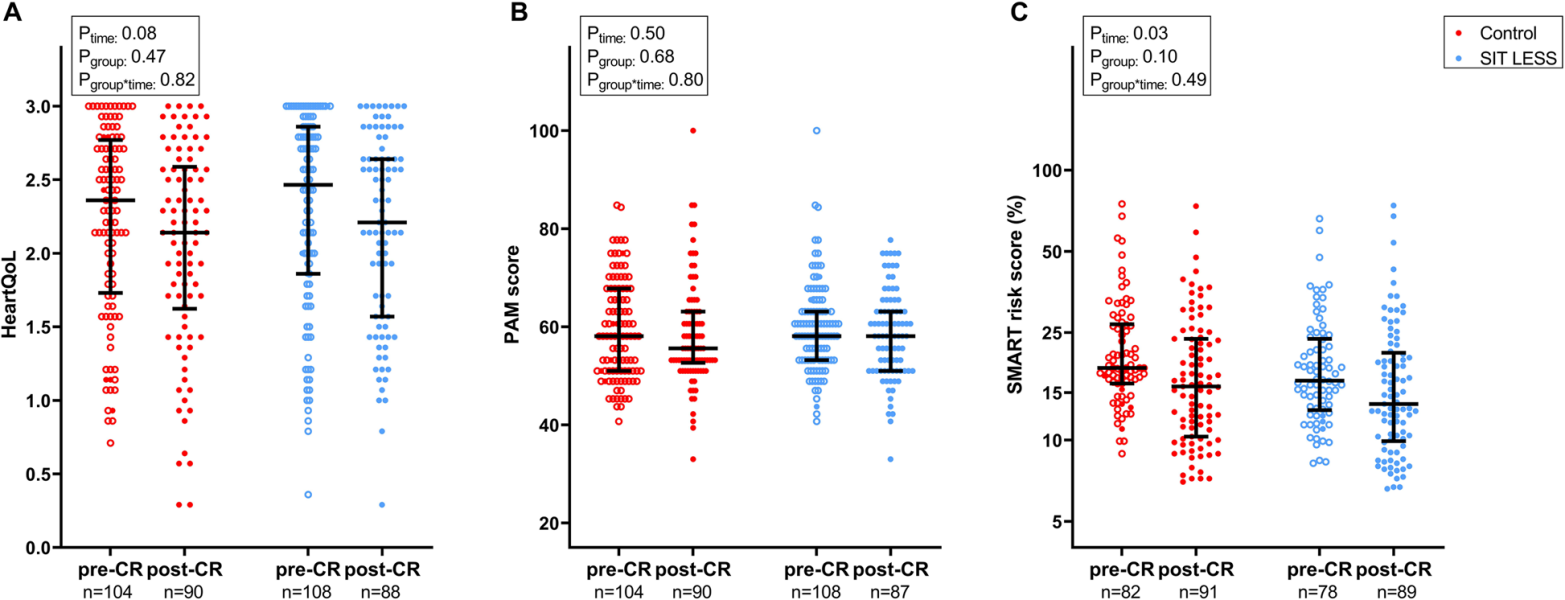
Quality of life, patients’ competencies for self-management and cardiovascular risk outcomes of the SIT LESS randomised clinical trial in patients with coronary artery disease pre- and post-cardiac rehabilitation (CR). Scatter plots with median and interquartile range of Heart quality of life (HeartQoL) score (panel **A**); Patient Activation Measure (PAM) score (panel **B**); and SMART cardiovascular risk score (panel **C**) compared between the control (in red) and the SIT LESS group (in blue). *P*-values are based on mixed model analysis

Time spent in LIPA, MVPA and step count increased from pre- to post-CR in both the SIT LESS (Δ LIPA: 1.3 (95% CI 0.9; 1.8); Δ MVPA: 0.3 (95% CI 0.2; 0.5) hours/day; Δ step count: 2969 (95% CI 2102; 3836) steps/day) and control group (Δ LIPA: 1.1 (95% CI 0.7; 1.5); Δ MVPA: 0.4 (95% CI 0.3; 0.5) hours/day; Δ step count: 3086 (95% CI 2180; 3992) steps/day). However, the magnitude of these improvements was not significantly different between SIT LESS and controls for LIPA time (0.2 (95% CI ─0.3; 0.8 h/day); MVPA time (0.0 (95% CI ─0.2; 0.1 h/day); and step count (− 117 (95% CI − 1367; 1134 steps/day) (Fig. 4). Changes in global HeartQoL (0.0 (95% CI ─0.2; 0.3), PAM score (─0.5 (95% CI ─4.8; 3.4) and SMART score (─0.1 (95% CI ─0.8; 0.5) did not differ between groups from pre- to post-CR (Fig. 5). Changes on physical and emotional HeartQoL subscales and within PAM-13 levels were not significantly different across the groups (Supplemental Table 4). Overall, patients graded study participation as valuable with a score of 8 [8-9] on a 10-point scale, which did not differ between SIT LESS and controls (*p* = 0.18).

## Discussion

Our randomised clinical trial in 212 patients with CAD participating in CR showed that SIT LESS did not induce significantly greater reductions in ST compared to controls, but delivery was feasible and a reduced odds of ST ≥ 9.5 h/day was observed. We also observed improvements of physical activity levels and reductions in the number of prolonged sedentary bouts and cardiovascular risk score from pre- to post-CR, but the change was comparable between groups. Finally, no changes were found regarding quality of life and patients’ competencies for self-management over 12 weeks of follow-up.

In the past decade, there has been increasing interest for behavioural change interventions to improve physical activity and reduce sedentary behaviour [9]. Replacement of ST with light- and/or moderate-to-vigorous physical activity has large potential to improve cardiovascular risk factors and survival [5, 7, 8]. Previous studies indicated that ST can be reduced in healthy adults (− 0.5 h/day) [33]; clinical populations (− 1.1 h/day) [34]; and mixed populations (− 0.4 to − 0.9 h/day) [35, 36]. However, large variation across studies exists in the magnitude of ST reductions, depending on the population of interest, type of intervention, and type of measurement (i.e. objective *versus* subjective assessments). Although absolute ST remains high after CR participation [14, 15], effective behavioural change interventions to reduce ST among patients with CAD are currently lacking.

SIT LESS was developed to specifically reduce ST in patients with CAD, co-created in close collaboration with end users (patients and nurse specialist) and tailored to the patients’ needs [23]. The difference in pre- to post-CR reduction in ST was ─0.4 (95% CI ─1.0; 0.3) hours/day in SIT LESS *versus* controls, thus lower than our intended effect size of − 0.5 h/day. A potential explanation for this finding may relate to sizeable reduction in ST of the control group (− 1.2 (95% CI − 1.7; − 0.8) hours/day), which was larger compared to findings from previous studies in comparable CR settings (− 0.4 (95% CI − 0.7; − 0.1) hours/day) [6, 13]. A meta-analysis including observations from multiple countries even found no significant reduction in ST following contemporary CR [12]. Since traditional CR has only marginal effects on changes in daily ST and does not include ST-focused elements, a crossover of elements (i.e. contamination) of the SIT LESS intervention to the control group may have occurred in our study. Indeed, patients could not be blinded to assignment of the intervention and nurses delivered traditional CR both with and without the SIT LESS intervention. Similarly, exchange of experiences among patients with CAD in the SIT LESS and control arm during the supervised group-based CR exercise sessions could not be prevented. Hence, it is plausible that some level of contamination has occurred, which could explain the substantial reduction in ST in the controls, thereby reducing the contrast between treatment arms. This concern can only be effectively addressed in a potential future cluster randomised controlled trial – a trial on a scale that was not realistic for a first evaluation of the SIT LESS intervention.

The observed reductions in ST in both groups could be clinically relevant. Isotemporal substitution analyses show that reducing 30 min of ST is associated with 2% lower risk of mortality and major adverse cardiovascular events [37] and 2–4% improvement in cardiovascular risk factors [38]. The relation between ST and (cardiovascular) mortality risk appears to be curvilinear, as the risk increases exponentially as daily ST increases [4]. Specifically, epidemiological evidence using device-based ST indicates that ST ≥ 9.5 h/day is associated with a significantly higher risk of death [4]. Patients in the SIT LESS group were informed about this threshold [23], leading to many patients setting their maximal ST goal (goal setting is part of the intervention strategy) at < 9.5 h/day. Post-CR, the proportion of patients with ST ≥ 9.5 h/day was significantly lower in those who followed SIT LESS compared to controls. Hence, the long-term health benefits of absolute reduction in ST as well as achieving an ST < 9.5 h/day should be evaluated in a future large-scale cluster randomised trial.

In line with the decrease in ST, the number of prolonged sedentary bouts decreased and time spent during LIPA and MVPA substantially increased from pre- to post-CR, but no additional effect of SIT LESS was found related to these parameters. Data regarding the direct response to vibrotactile feedback were not available, although this information could have been useful for a fidelity check of the activity tracker. Nevertheless, if these acute improvements in physical activity and prolonged sedentary bouts can be maintained after study participation, these changes are likely being accompanied by beneficial long-term health effects [4, 39, 40]. Previous work has shown that the total volume of physical activity, regardless of the intensity, is associated with mortality risk reductions [4]. Small improvements in LIPA (+ 35 min/day) and MVPA (+ 5 min/day) result in risk reductions for mortality [4], whereas regular breaking-up of sedentary time is associated with improvements in post-prandial glucose levels, triglycerides, insulin resistance and adiposity measures [39, 40]. Hence transitioning towards a physically active lifestyle is expected to yield long-term health benefits in patients with CAD.

Quality of life did not change across the study period, which is in contrast to previous observations [27]. An explanation for the lack of quality of life improvement may relate to the timing of HeartQoL during index hospitalisation, as this questionnaire assesses the quality of life in the previous four weeks which was pre-CAD diagnosis for most patients. Patients’ competencies for self-management did not improve following CR. At post-CR, absolute PAM-13 scores were lower compared to other patients with chronic illness [41], but most patients with CAD were at the level where they have adopted behaviours to support health or take action. Nevertheless, further improvement of self-efficacy should be targeted in future CR programmes, as this is an important factor for the success of behaviour change interventions [42].

Strengths of our study include the device-based assessment of ST, co-creation of SIT LESS, a substantive trial for a first test of the SIT LESS intervention, and the low dropout rates supporting the feasibility and acceptability of SIT LESS. Feasibility was also reflected by the high level of adherence to the intervention and activity tracker; the successful implementation at two different centres in clinical practice and the high ratings of patients for participating in the study. Our study also has some limitations. First, due to the nature of the intervention, investigators, nurse specialists and patients were not blinded for the treatment. This may have contributed to contamination bias from the SIT LESS intervention to the control group and a subsequent underestimation of the true difference between SIT LESS and controls. Unfortunately, indicative measures for contamination, e.g. knowledge regarding ST-associated health risks were not collected, so the level of contamination could not be quantified. Second, it remains unknown whether the overall decrease in ST is sustainable over time and how this relates to future clinical endpoints. Therefore, larger, cluster randomised trials with longer-term follow-up are warranted, also including assessment of cost-effectiveness and sufficiently powered to detect improvements in event-free survival of patients with CAD.

### Practical implications

This study demonstrates that the nurse-delivered SIT LESS behaviour change intervention appears feasible, acceptable and potentially beneficial to reduce time spent sedentary among patients with CAD. A larger, cluster randomised trial is warranted to provide a more accurate estimate of its effects on sedentary time and clinical outcomes. Reducing sedentary behaviour is a promising target in preventive cardiology and may improve habitual physical activity of cardiovascular disease patients beyond supervised exercise training sessions. Personalised behaviour change interventions that are supported by technology-based programmes and supplemented with (digital) coaching may become the new standard of future CR programmes.

## Conclusions

In conclusion, the SIT LESS RCT results indicate that among patients with coronary artery disease participating in cardiac rehabilitation, the SIT LESS intervention did not induce significantly greater reductions in sedentary time compared to controls, but delivery was feasible and resulted in a reduced odds of a sedentary time ≥ 9.5 h/day. Hence, the results of our study appear sufficiently promising for conducting a future large-scale cluster randomised trial of SIT LESS.

## Supplementary Information

**Additional file 1: Supplemental Figure 1.** Forest plot with stratified mixed model analyses on the impact of SIT LESS on changes in sedentary time from pre- to post-CR, the black squares indicate the estimates and the lines represent the 95% confidence intervals of the estimate. CABG: coronary artery bypass grafting; NSTEMI: non-ST-elevation myocardial infarction; PCI: Percutaneous Coronary Intervention; STEMI: ST-elevation myocardial infarction; (U)AP: (unstable) angina pectoris.

**Additional file 2: Supplemental Figure 2.** Longitudinal sedentary time (panel A) and prolonged sedentary bouts (≥ 30 min) (panel B) during SIT LESS based on the activity tracker data (SIT LESS group). Data are presented as median with interquartile range. The dashed line in panel A represents the upper-limit of normal daily sedentary time (9.5 hours per day). *P*-values are based on mixed model analysis to assess changes in sedentary behaviour during the intervention period using random intercepts with time as continuous variable, ranging from week 1 to week 12.

**Additional file 3: Supplemental Table 1.** Cardiac rehabilitation characteristics.

**Additional file 4: Supplemental Table 2.** SIT LESS intervention characteristics.

**Additional file 5: Supplemental Table 3.** Reasons for premature discontinuation SIT LESS activity tracker.

**Additional file 6: Supplemental Table 4.** Quality of life, self-efficacy and cardiovascular risk score.

**Additional file 7: Supplemental Document 1.** SIT LESS manual.

**Additional file 8: Supplemental Document 2.** CONSORT checklist.

**Additional file 9: Supplemental Document 3.** TIDieR.

## Data Availability

Data from the SIT LESS randomised clinical trial are available upon reasonable request via the corresponding author.

## Abbreviations

CABG: Coronary artery bypass grafting
CAD: Coronary artery disease
CR: Cardiac rehabilitation
LIPA: Light-intensity physical activity
MVPA: Moderate-to-vigorous intensity physical activity
NSTEMI: Non-ST-elevated myocardial infarction
NYHA: New York Heart Association
SIT LESS: Sedentary Behaviour Intervention as a Personalised Secondary Prevention Strategy
ST: Sedentary time
STEMI: ST-elevated myocardial infarction
