# Supplementary material for: Effectiveness of an intervention to reduce sedentary behaviour as a personalised secondary prevention strategy for patients with coronary artery disease: main outcomes of the SIT LESS randomised clinical trial
Source: Int J Behav Nutr Phys Act. 2023 Feb 14;20:17. doi: 10.1186/s12966-023-01419-z (PMC9927064; doi:10.1186/s12966-023-01419-z)

Subgroup	Control pre-CR (n)	Control post-CR(n)	SIT LESS pre-CR (n)	SIT LESS post-CR(n)
Male	79	71	79	69
Female	21	22	24	21
Age ⩽63	46	42	48	44
Age > 63	54	51	55	46
Employed	43	42	49	50
Unemployed	52	50	42	39
Living environment - urban	31	30	22	23
Living environment - rural	20	20	29	28
Living environment - transition	45	43	40	38
Education - high	29	29	37	38
Education - middle	39	37	36	33
Education - low	27	26	18	18
STEMI	32	29	28	27
NSTEMI	43	39	54	46
(U)AP	25	25	21	17
CABG	27	27	27	22
PCI	61	56	58	51
**Total**	**100**	**93**	**103**	**90**

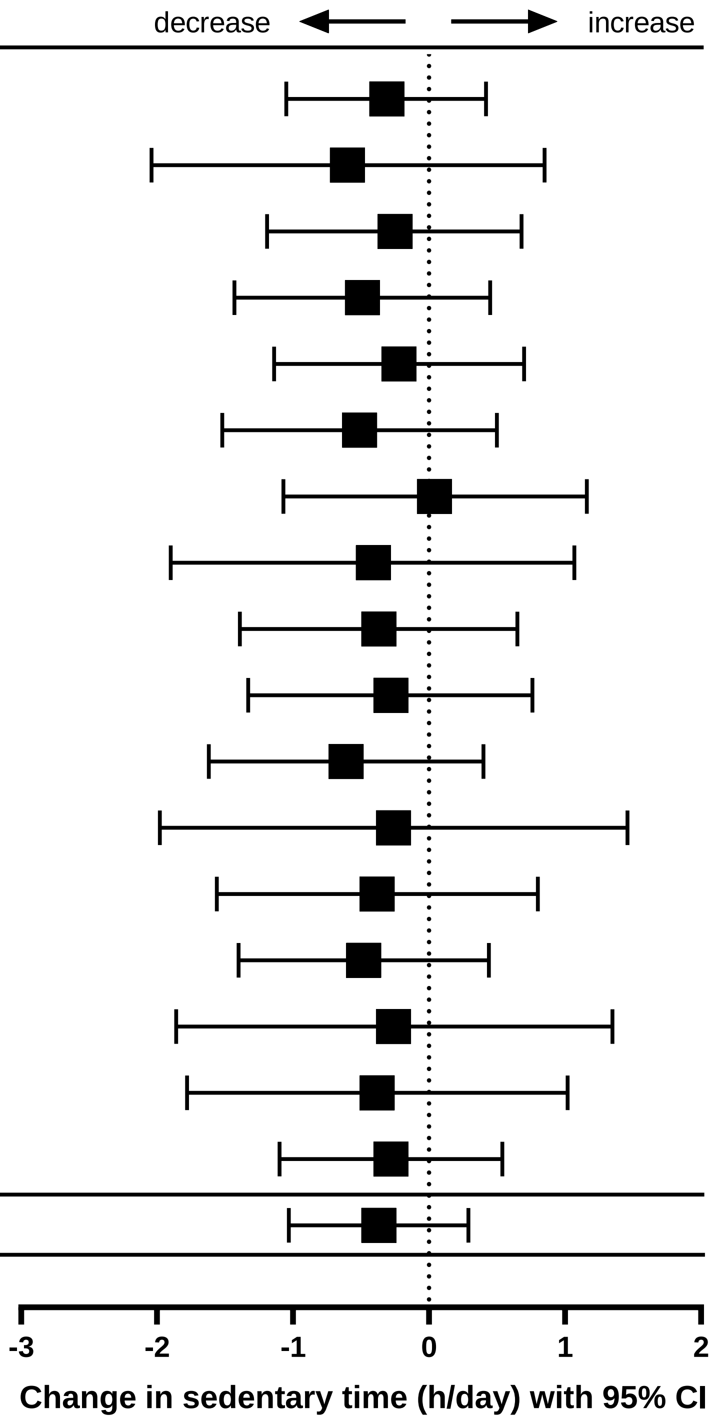

Supplement: Supplementary file 1 — Additional file 1: Supplemental Figure 1. Forest plot with stratified mixed model analyses on the impact of SIT LESS on changes in sedentary time from pre- to post-CR, the black squares indicate the estimates and the lines represent the 95% confidence intervals of the estimate. CABG: coronary artery bypass grafting; NSTEMI: non-ST-elevation myocardial infarction; PCI: Percutaneous Coronary Intervention; STEMI: ST-elevation myocardial infarction; (U)AP: (unstable) angina pectoris. [file 12966_2023_1419_MOESM1_ESM.pdf]
