# Supplementary figures and images for: Effectiveness of an intervention to reduce sedentary behaviour as a personalised secondary prevention strategy for patients with coronary artery disease: main outcomes of the SIT LESS randomised clinical trial
Source: Int J Behav Nutr Phys Act. 2023 Feb 14;20:17. doi: 10.1186/s12966-023-01419-z (PMC9927064; doi:10.1186/s12966-023-01419-z)

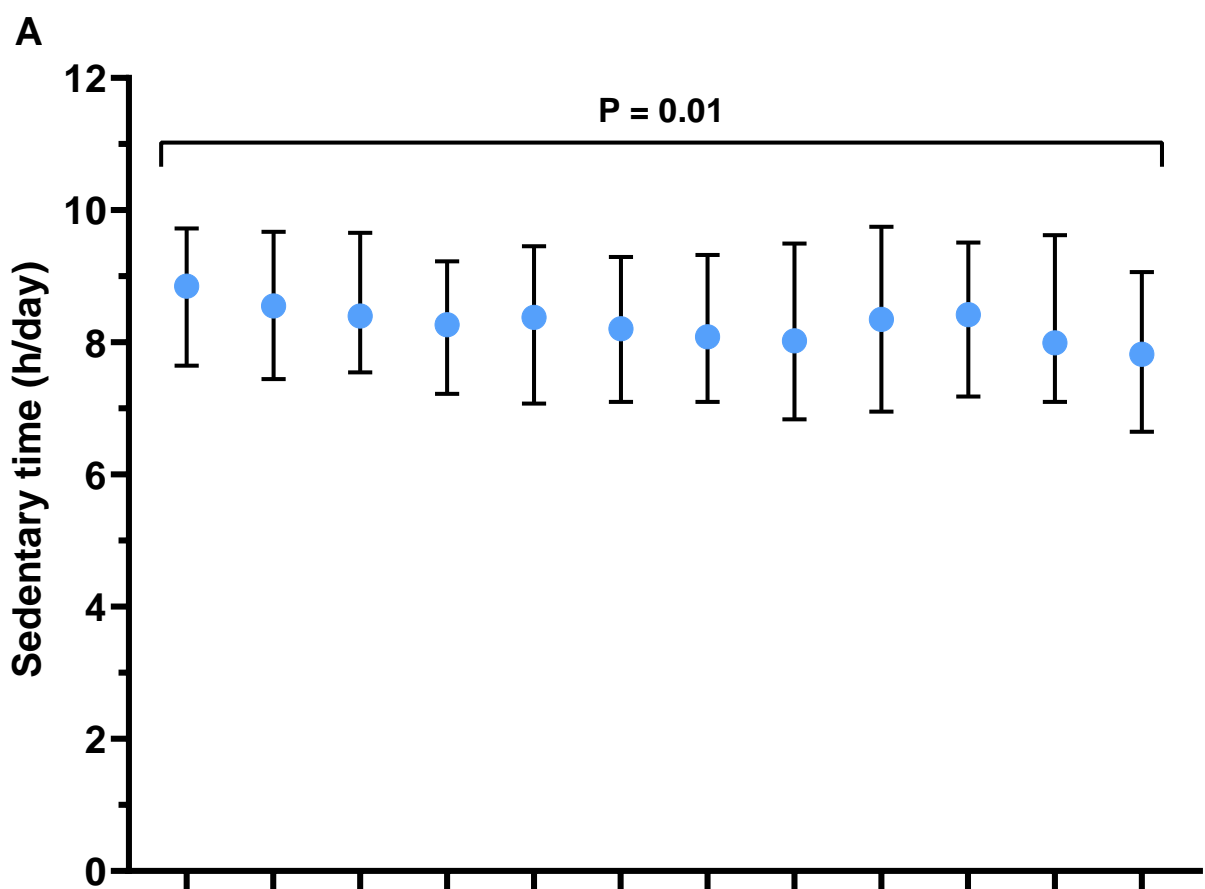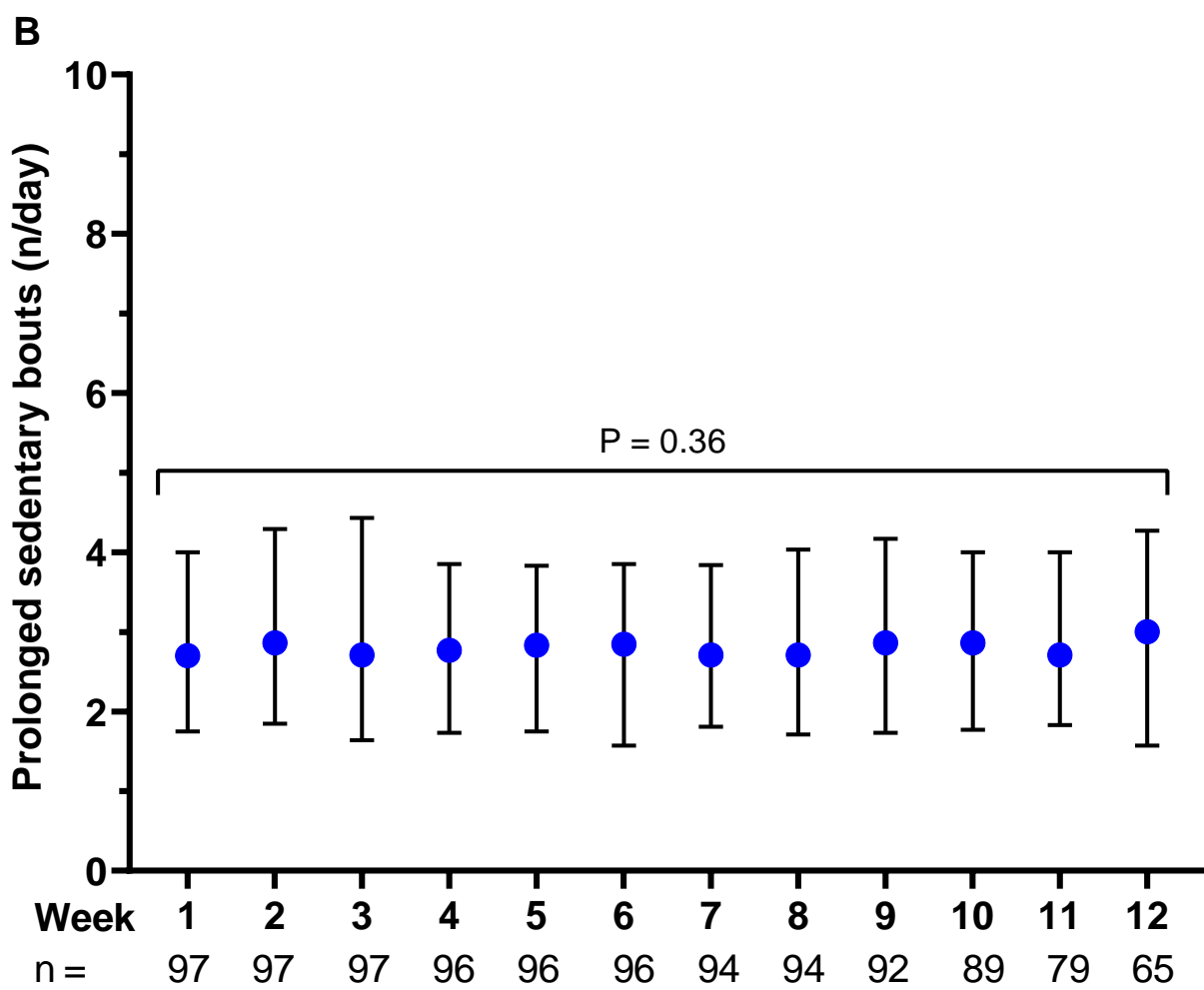

Supplement: Supplementary file 2 — Additional file 2: Supplemental Figure 2. Longitudinal sedentary time (panel A) and prolonged sedentary bouts (≥ 30 min) (panel B) during SIT LESS based on the activity tracker data (SIT LESS group). Data are presented as median with interquartile range. The dashed line in panel A represents the upper-limit of normal daily sedentary time (9.5 hours per day). P-values are based on mixed model analysis to assess changes in sedentary behaviour during the intervention period using random intercepts with time as continuous variable, ranging from week 1 to week 12. [file 12966_2023_1419_MOESM2_ESM.pdf]
