# Supplementary material for: Effectiveness of an intervention to reduce sedentary behaviour as a personalised secondary prevention strategy for patients with coronary artery disease: main outcomes of the SIT LESS randomised clinical trial
Source: Int J Behav Nutr Phys Act. 2023 Feb 14;20:17. doi: 10.1186/s12966-023-01419-z (PMC9927064; doi:10.1186/s12966-023-01419-z)
Supplement: Supplementary file 3 — Additional file 3: Supplemental Table 1. Cardiac rehabilitation characteristics. [file 12966_2023_1419_MOESM3_ESM.docx]

**Supplemental Table 1.** Cardiac rehabilitation characteristics.

|  | | Total population (n=212) | Missing values (n (%)) | SIT LESS group (n=108) | Control group (n=104) |
| --- | --- | --- | --- | --- | --- |
| Intake consult CR (n (%)) | | 211 (100%) | 0 (0%) | 107 (99%) | 104 (100%) |
| Exit consult CR (n (%)) | | 197 (96%) | 7 (3%) | 98 (95%) | 99 (97%) |
| CR not completed (n (%)) | | 8 (4%) | 7 (3%) | 5 (5%) | 3 (3%) |
| Exercise training at CR (yes) (n (%)) | | 171 (88%) | 17 (8%) | 85 (88%) | 86 (88%) |
|  | Supervised exercise sessions (n) | 11 [9-11] | 0 (0%) | 11 [9-11] | 11 [9-12] |

Data are displayed as n (%) for categorical variables and or median [interquartile range] for continuous variables. CR: cardiac rehabilitation.
