# Supplementary material for: Effectiveness of an intervention to reduce sedentary behaviour as a personalised secondary prevention strategy for patients with coronary artery disease: main outcomes of the SIT LESS randomised clinical trial
Source: Int J Behav Nutr Phys Act. 2023 Feb 14;20:17. doi: 10.1186/s12966-023-01419-z (PMC9927064; doi:10.1186/s12966-023-01419-z)
Supplement: Supplementary file 4 — Additional file 4: Supplemental Table 2. SIT LESS intervention characteristics. [file 12966_2023_1419_MOESM4_ESM.docx]

**Supplemental Table 2.** SIT LESS intervention characteristics.

|  | | | SIT LESS group (n=108) | Missing values (n (%)) |
| --- | --- | --- | --- | --- |
| CR - SIT LESS coaching consultations | | |  |  |
|  | Intake consult CR + SIT LESS (n (%)) | | 107 (99%) | 0 (0%) |
|  | Interim consult SIT LESS (n (%)) | | 101 (94%) | 0 (0%) |
|  | Exit consult CR + SIT LESS (n (%)) | | 97 (90%) | 0 (0%) |
|  | CR - SIT LESS coaching not completed (n (%)) | | 11 (10%) | 0 (0%) |
| SIT LESS - telephone coaching | | |  |  |
|  | SIT LESS telephone coaching sessions total intervention period (n (%)) | | 7 [6-8] | 0 (0%) |
| Duration intervention period (days)* | | | 89 (±13) | 0 (0%) |
| SIT LESS - activity tracker* | | |  |  |
|  | Number of days with ≥ 10 h wear time (days) | | 71 (±19) | 0 (0%) |
|  | Adherence (%) | | 84 [72-94] | 0 (0%) |
|  | Premature discontinuation activity tracker use (n (%)) | | 11 (11%) | 0 (0%) |
|  |  | Before interim consult (n (%)) | 3 (3%) | 0 (0%) |
|  |  | After interim consult (n (%)) | 8 (8%) | 0 (0%) |

Data are presented as n (%), mean (± standard deviation) or median [interquartile range]. CR: Cardiac rehabilitation.
*patients who did not complete CR - SIT LESS coaching were excluded.
