# Supplementary material for: Effectiveness of an intervention to reduce sedentary behaviour as a personalised secondary prevention strategy for patients with coronary artery disease: main outcomes of the SIT LESS randomised clinical trial
Source: Int J Behav Nutr Phys Act. 2023 Feb 14;20:17. doi: 10.1186/s12966-023-01419-z (PMC9927064; doi:10.1186/s12966-023-01419-z)
Supplement: Supplementary file 5 — Additional file 5: Supplemental Table 3. Reasons for premature discontinuation SIT LESS activity tracker. [file 12966_2023_1419_MOESM5_ESM.docx]

**Supplemental Table 3.** Reasons for premature discontinuation SIT LESS activity tracker.

|  | | | SIT LESS group without dropouts (n=97) |
| --- | --- | --- | --- |
| Premature discontinuation activity tracker use (n (%)) | | | **11 (11%)** |
|  | Before interim consult (n (%)) | | 3 (3%) |
|  | | Stress and obsessive behaviour | 1 (1%) |
|  | | Discontent about accuracy | 1 (1%) |
|  | | Illness | 1 (1%) |
|  | After interim consult (n (%)) | | 8 (8%) |
|  | | Discontent about accuracy | 3 (3%) |
|  | | Personal circumstances | 1 (1%) |
|  | | Technical reasons | 2 (1%) |
|  | | Unknown | 2 (2%) |

Data are presented as n (%).
