# Supplementary material for: Effectiveness of an intervention to reduce sedentary behaviour as a personalised secondary prevention strategy for patients with coronary artery disease: main outcomes of the SIT LESS randomised clinical trial
Source: Int J Behav Nutr Phys Act. 2023 Feb 14;20:17. doi: 10.1186/s12966-023-01419-z (PMC9927064; doi:10.1186/s12966-023-01419-z)
Supplement: Supplementary file 6 — Additional file 6: Supplemental Table 4. Quality of life, self-efficacy and cardiovascular risk score. [file 12966_2023_1419_MOESM6_ESM.docx]

**Supplemental Table 4.** Quality of life, self-efficacy and cardiovascular risk score.

|  | | Total population  (n=212) | | Missing values (n (%)) | | SIT LESS group  (n=108) | | Control group  (n=104) | | P-value |
| --- | --- | --- | --- | --- | --- | --- | --- | --- | --- | --- |
|  | | **pre-CR** | **post-CR** | **pre-CR** | **post-CR** | **pre-CR** | **post-CR** | **pre-CR** | **post-CR** |  |
| HeartQoL [range 0-3] | |  |  | 0 (0%) | 34 (16%) |  |  |  |  |  |
|  | Global | 2.4 [1.8-2.8] | 2.2 [1.6-2.6] |  |  | 2.5 [1.9-2.9] | 2.2 [1.6-2.6] | 2.4 [1.7-2.8] | 2.1 [1.6-2.6] | 0.82 |
|  | Physical | 2.5 [1.5-3.0] | 2.2 [1.4-2.7] |  |  | 2.6 [1.8-3.0] | 2.4 [1.4-2.8] | 2.6 [1.8-2.9] | 2.1 [1.4-2.7] | 0.57 |
|  | Emotional | 2.6 [1.8-2.9] | 2.5 [1.7-3.0] |  |  | 2.5 [1.8-3.0] | 2.5 [1.8-3.0] | 2.3 [1.5-3.0] | 2.5 [1.5-3.0] | 0.50 |
| PAM-13 | |  |  | 0 (0%) | 35 (17%) |  |  |  |  | 0.67 |
|  | Level 1: ≤ 47.0 points (n (%)) | 17 (8%) | 17 (10%) |  |  | 6 (6%) | 8 (9%) | 11 (11%) | 9 (10%) |  |
|  | Level 2: 47.1-55.1 points (n (%)) | 57 (27%) | 54 (31%) |  |  | 26 (24%) | 23 (26%) | 31 (30%) | 31 (34%) |  |
|  | Level 3: 55.2-67.0 points (n (%)) | 89 (42%) | 75 (42%) |  |  | 55 (51%) | 40 (46%) | 34 (33%) | 35 (39%) |  |
|  | Level 4: ≥67.1 points (n (%)) | 49 (23%) | 31 (18%) |  |  | 21 (19%) | 16 (18%) | 28 (27%) | 15 (17%) |  |
| SMART cardiovascular risk score (%) | | 18 [15-25] | 15 [10-23] | 52 (25%) | 32 (15%) | 17 [13-24] | 14 [10-21] | 19 [16-27] | 16 [10-24] | 0.49 |

Data are displayed as n (%) for categorical variables and as mean (± standard deviation) or median [interquartile range] for continuous variables. P-values represent the difference between SIT LESS and control group in the change from pre- to post-CR, based on mixed model analyses (HeartQoL and SMART score) or the difference at post-CR between SIT LESS and control group, based on the chi-square test (PAM-13). CR: cardiac rehabilitation; HeartQoL: Heart Quality of Life; PAM-13: 13-item Patient Activation Measure (self-efficacy score).
