# Supplementary material for: Effectiveness of an intervention to reduce sedentary behaviour as a personalised secondary prevention strategy for patients with coronary artery disease: main outcomes of the SIT LESS randomised clinical trial
Source: Int J Behav Nutr Phys Act. 2023 Feb 14;20:17. doi: 10.1186/s12966-023-01419-z (PMC9927064; doi:10.1186/s12966-023-01419-z)
Supplement: Supplementary file 7 — Additional file 7: Supplemental Document 1. SIT LESS manual. [file 12966_2023_1419_MOESM7_ESM.docx]

**Supplemental Document 1.**

**To:**

**Effectiveness of a** **Sedentary Behaviour Intervention to reduce sedentary behaviour as a Personalised Secondary Prevention Strategy (SIT LESS) for patients with Coronary Artery Disease: main outcomes of the SIT LESS randomised clinical trial**

B B.M.A. van Bakel, MD^1^; S.H. Kroesen, MSc^1^; E.A. Bakker, PhD^1^; R.V. van Miltenburg, BSc^1^; A. Günal, MD^2^; A. Scheepmaker, MD^2^; W.R.M. Aengevaeren, MD, PhD^3^; F.F. Willems, MD, PhD^3^; R. Wondergem, PhD^4,5,6^; M.F. Pisters, PhD^4,5,6^; M. de Bruin, PhD^7^; M.T.E. Hopman, MD, PhD^1^; D.H.J. Thijssen, PhD^1,8^; T.M.H. Eijsvogels, PhD^1^

**Affiliations:**

1. Radboud university medical center, Radboud Institute for Health Sciences, Department of Physiology, Nijmegen, The Netherlands
2. Bernhoven hospital, Department of Cardiology, Uden, The Netherlands
3. Rijnstate hospital, Department of Cardiology, Arnhem, The Netherlands
4. Utrecht University, University Medical Center Utrecht Brain Center, Department of Rehabilitation, Physical Therapy Science and Sport, Utrecht, The Netherlands
5. Research Group Empowering Healthy Behaviour, Department of Health Innovations and Technology, Fontys University of Applied Sciences, Eindhoven, The Netherlands
6. Center for Physical Therapy Research and Innovation in Primary Care, Julius Health Care Centers, Utrecht, the Netherlands
7. Radboud university medical center, Radboud Institute for Health Sciences, Department of IQ healthcare, Nijmegen, The Netherlands
8. Research Institute for Sports and Exercise Sciences, Liverpool John Moores University, Liverpool, United Kingdom

**Corresponding author:**Thijs M.H. Eijsvogels, PhD, Radboud university medical center, Dept. of Physiology, P.O. Box 9101, 6500 HB, Nijmegen, The Netherlands. E-mail: Thijs.Eijsvogels@radboudumc.nl Telephone: (+31) (0)24 36 14273 Fax: (+31) (0)24 3668340

**SIT LESS intervention manual**


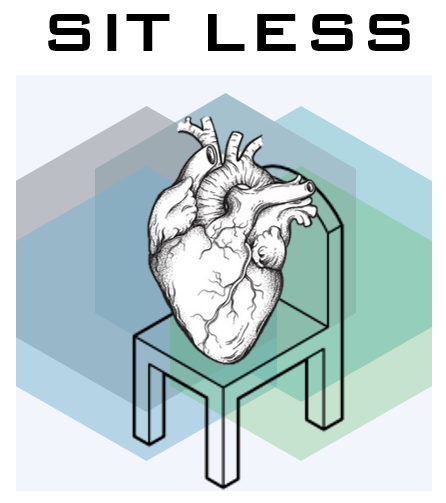
The SIT LESS manual is used by the nurse specialist during three face-to-face consultations for SIT LESS coaching. The nurse specialists received a comprehensive and accredited training course in using this manual during the SIT LESS consultations. During the first consultations, steps 1 to 7 are discussed. During the second and third consultation step 8 is discussed and, if indicated, previous steps of the manual can be repeated.


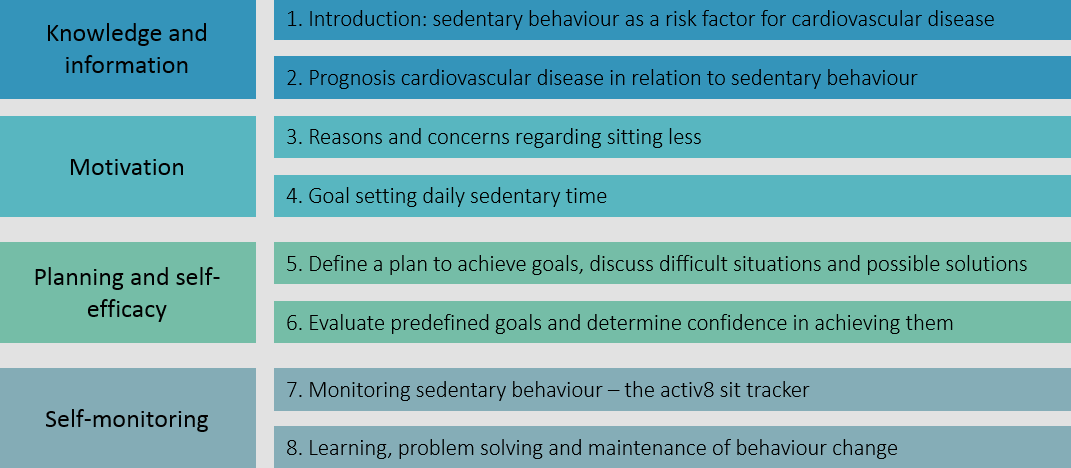


SIT LESS programme –

Intervention manual

# Introduction: sedentary behaviour as a risk factor for cardiovascular disease

AIM: Introduce prolonged sitting as a risk factor for the progression of cardiovascular disease and inform the patient about the facilitating role of cardiac rehabilitation in targeting risk factors. Patient understands prolonged sitting as one of the risk factors for cardiovascular disease and knows that this is the point of attention of this additional part of cardiac rehabilitation.

CONTENT: In this step, knowledge about risk factors of cardiovascular disease is identified, misconceptions are addressed and prolonged sitting as a point of attention of this part with the aid of visual material is introduced.

1. Risk factors

Recently, you were diagnosed with heart problems. You possible underwent surgery and/or got medicines to treat your condition. Besides good medical care, a healthy lifestyle can help to lower the chance of new heart problems. Factors that increase the chance of developing new heart problems or early mortality are called risk factors: [show the patient the Figure below]


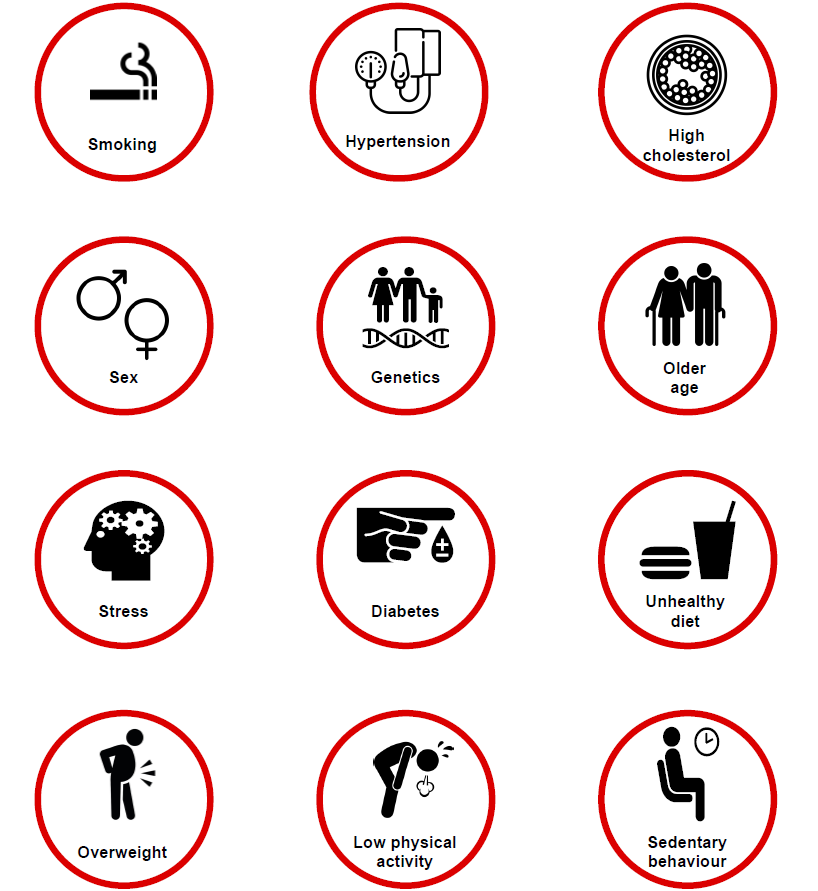


Explanation by the Figure S2-1A above:
[Possibly connect to the risk factors that were addressed earlier in the consultation.]
*1. There are many factors that play a part in the risk of developing cardiovascular diseases as you can see. Some of these factors are non-modifiable (genetic factors like sex, genetic predisposition, older age) and others are modifiable. One of the modifiable factors is the amount of physical activity and sedentary behaviour.
2. What do you know about the effects of prolonged sitting on the risk of developing cardiovascular diseases?
3. Connect to the mentioned factors [Good that you are familiar with xxx and xxx as important effects of prolonged sitting]
4. Correct possible misconceptions [You mentioned xxx as an effect of prolonged sitting, however xxx has no influence on the risk of developing cardiovascular diseases/xxx increases the risk of cardiovascular diseases via xxx]
5. Enforce the mentioned effects [Indeed, prolonged sitting increases the risk of developing cardiovascular diseases substantially]
6. Ask the patient what he/she thinks about that [How do you feel about that? What does that mean to you?]*

1. Cardiac Rehabilitation

Cardiac rehabilitation assists you with recovering after your cardiovascular event, improving your condition and getting confidence in your own body again. We work together on a healthier lifestyle where we try to decrease the possible risk factors as much as possible. That is why cardiac rehabilitation consists of different components. Ultimately, it is a program tailored to the individual. Every person is unique!

In this specific part within the cardiac rehabilitation, we focus on an important risk factor: prolonged sitting.

May I tell you something about that?

# Prognosis cardiovascular disease in relation to sedentary behaviour

AIM: The patient understands the role of sitting less in relation to cardiovascular diseases. The patient is aware of the benefits of sitting less and interrupting sitting. The patient does also understand the benefits of more physical activity. There are no misconceptions anymore.

CONTENT: In this step, the knowledge about much and prolonged sitting and the consequences on health are discussed. Misconceptions are addressed and complex concepts are shown in a simple visual way. The benefits of sitting less and interruption of prolonged sitting are clear. By discussing the relation between the daily amount of physical activity and health, the whole physical activity spectrum is addressed. Sitting less + more physical activity (instead) leads to maximal health benefits.

A. Sedentary behaviour

What do we mean by sedentary behaviour? Sedentary behaviour consists of activities with a low energy expenditure while sitting or lying. We sit more than we think and are mostly unaware of that, see the Figure below:


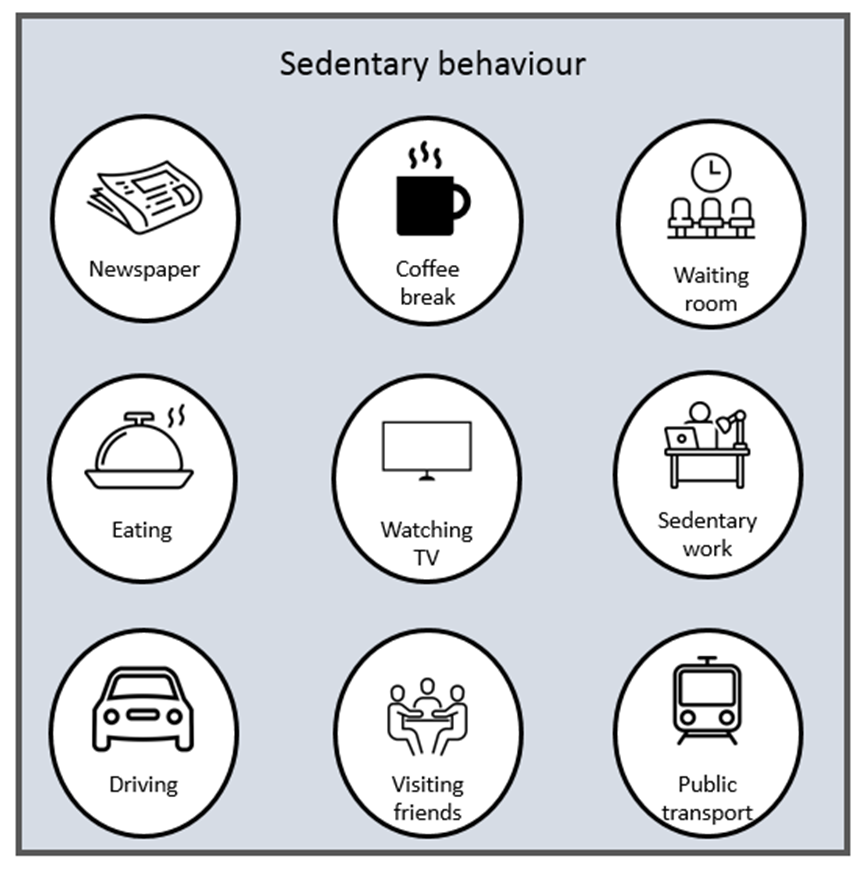


Explanation by the Figure S2-2A1 above:
*Our life consists of many sitting moments, for example when reading the paper, drinking coffee, waiting for the train, bus or at the pharmacy, while eating, watching TV on the couch, doing administrative tasks, driving, when someone is visiting, while travelling. The daily sitting duration can unconsciously quickly at up.*

*Ask if the patient recognizes sitting moments in their daily life: [Do you recognize those sedentary moment in you daily life?]*

*Below, there is an example of the daily schedule with sitting moment of a heart patient. We have identified the sedentary situations on a regular week and workday:*


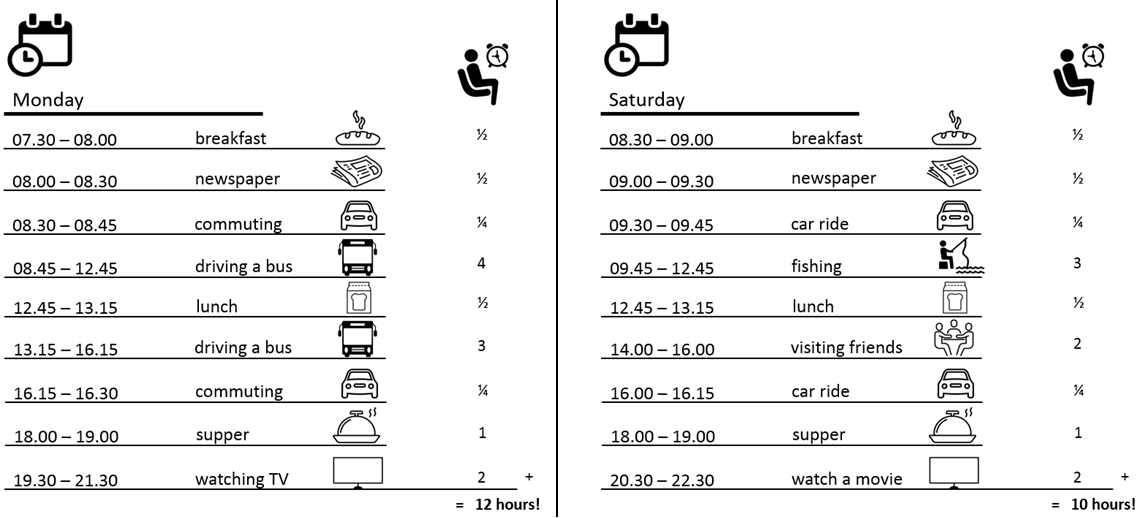


Explanation by the Figure S2-2A2 above:
*On the left side, you can see all the sedentary behaviour of a cardiovascular disease patient during a weekday as a bus driver. First, eating breakfast and reading the newspaper for half an hour. Thereafter, taking the car to work and sitting while driving the bus for work. There is a seated lunch break in between. The patient drives home again by car, a seated supper and eventually 2 hours of watching TV on the couch. Quite a normal day for this patient, however, all the sitting moments add up to a total of 12 hours on one single day.*

*On the right side, you can see the sedentary behaviour on a general weekend day. On Saturday, the day starts with a seated breakfast and reading the newspaper. Thereafter, the patient takes the car to go fishing. After lunch and visiting friends in the neighborhood, the patient drives home again for supper. At the end of the day, the patient watches a movie on the couch. In total 10 hours of sitting.*

*As you can see, you easily spend many hours a day sitting.*

*Ask the patient how he thinks about it: [How do you see your daily amount of sitting in your daily routine?] You can monitor you sitting moments on a weekday and weekend day the upcoming weeks if you want. To do so, you can use the empty agenda. We can discuss the results during the next consultation. [May this be something for you?]*


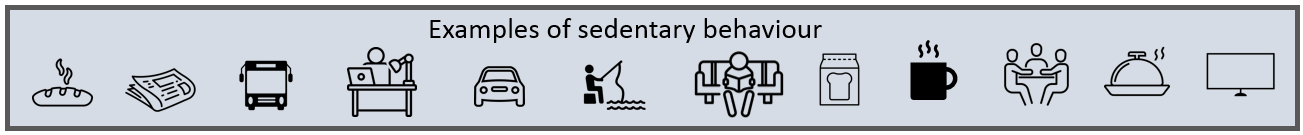

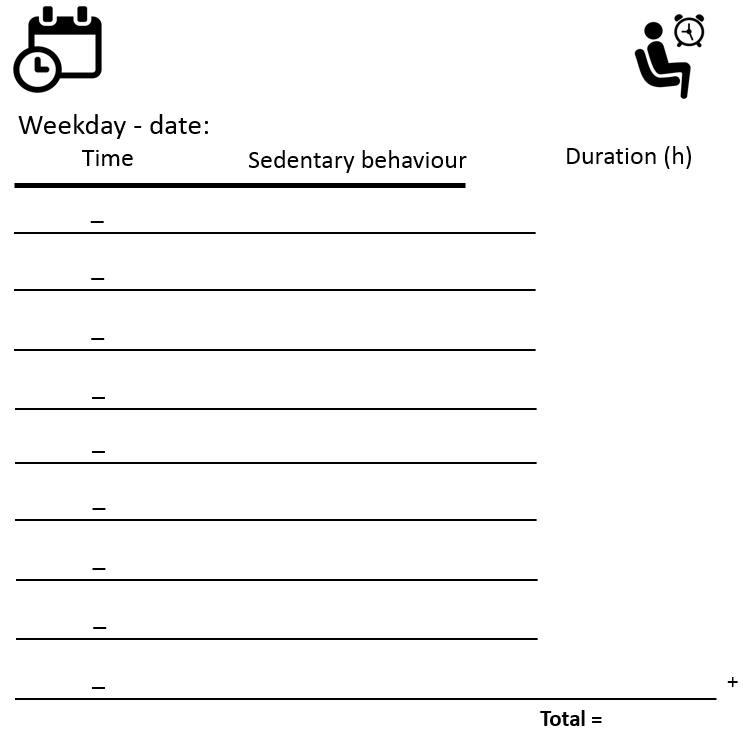

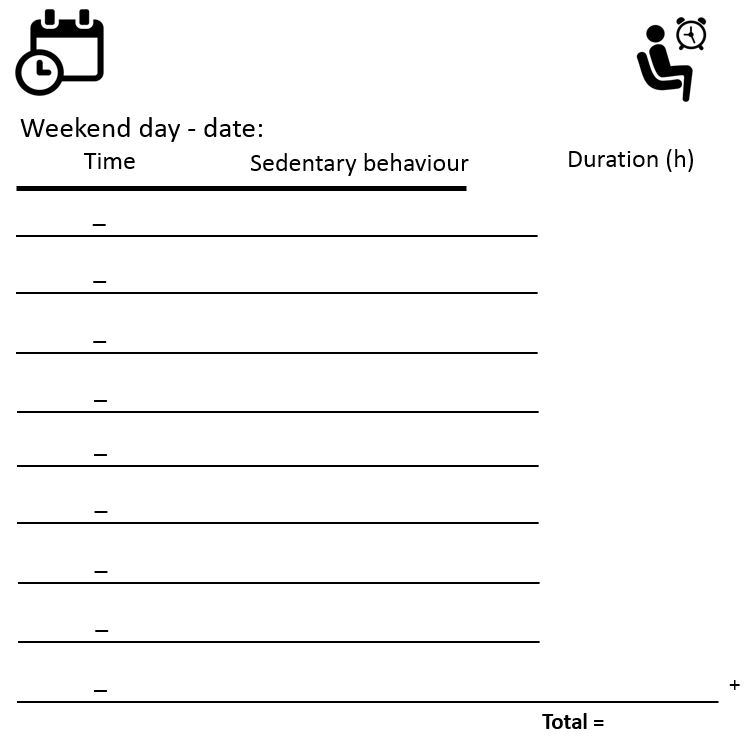


B. Detrimental health consequences of sedentary behaviour

After the cardiovascular event, you may have the feeling that your body has to recover. Consequently, sitting time could further increase. Why is that a problem and why is prolonged sitting unhealthy? Prolonged sitting as a detrimental effect on the body in many ways. You can see the health effects of prolonged sitting in the Figure below:


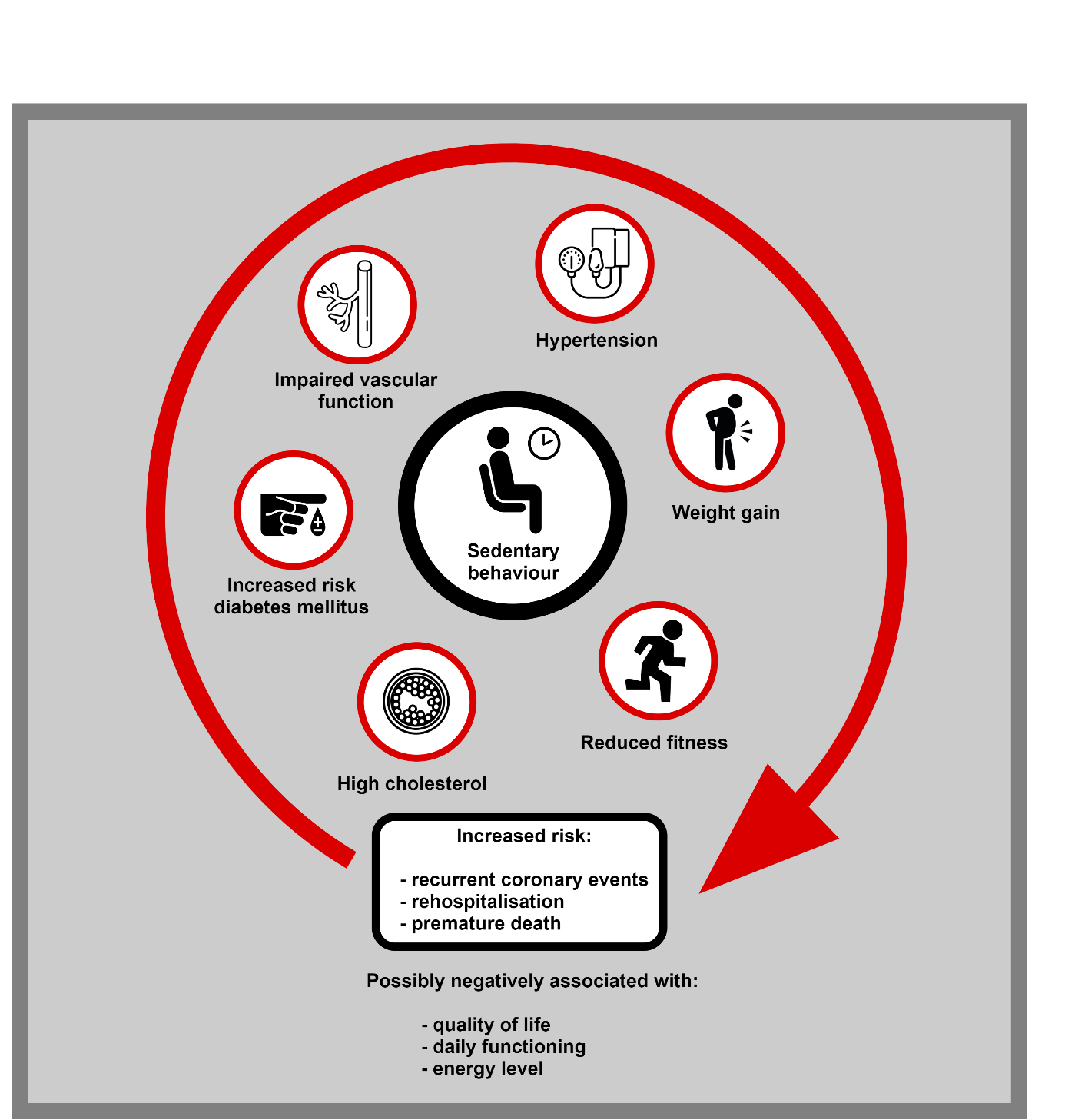


Explanation by the Figure S2-2B above:
*People who are highly sedentary, have increased cholesterol levels, an increased risk of developing diabetes mellitus, a higher blood pressure, are more often overweight and have a reduced fitness. That is why the chance of developing new heart problems, a hospital admission or mortality increases. Besides, prolonged sitting can lead to a lower quality of life, lower energy levels and doing less in daily life. We can help you to prevent these detrimental effects. Sitting less increases your chances on a longer and healthier life!*

1. Sitting less and regularly stand up improves health!

So, sitting less has beneficial effects on your health. By breaking up sitting regularly, at least every 30 minutes, and standing for a moment, stretching the legs or walk you get moving and lower the total sedentary time. Not only by reducing the total sedentary time, but also by standing up more often and breaking up sitting you can improve your health! How that works can be see in the Figure below. Shall we have a look at it together?


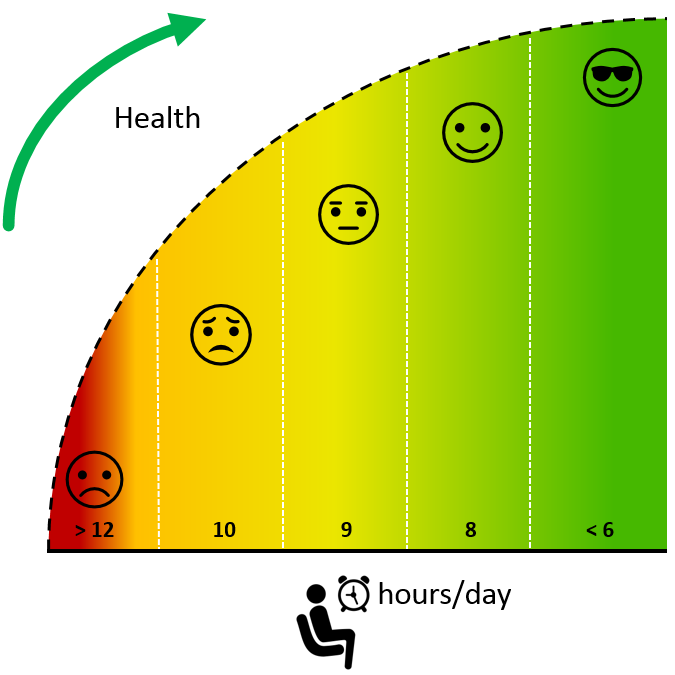

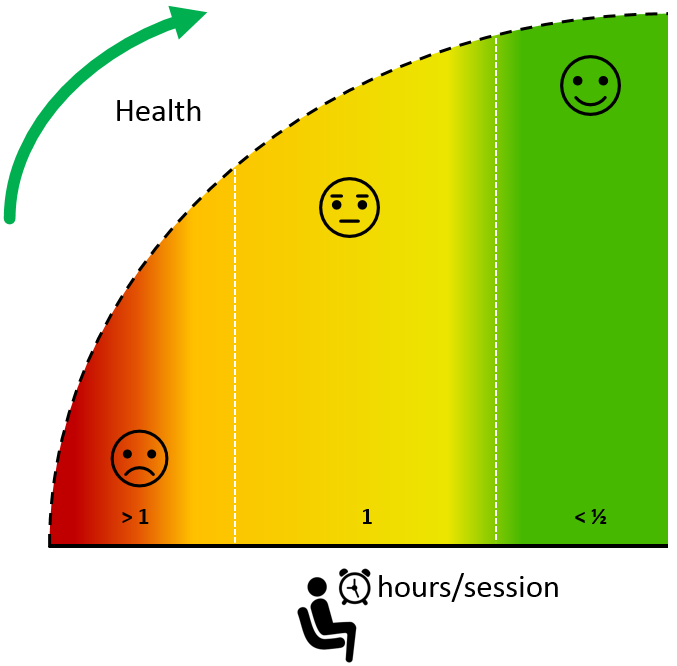


Explanation by the Figure S2-2C above:
*Let’s have a look at the left side first. Here you can see [point out] the total sitting hours a day from left to right. The hours per day vary from very high (more than 12 hours a day) to very little (less than 6 hours a day). From below to the top the health level is depicted. The higher on the graph, the better your health. Particilarly when you are in the risk zone at the moment (sitting 10 hours per day or more), you can improve your health substantially by reducing daily sedentary time with 1 hour. That is why the graph has the shape of a bow; the largest improvements to your health find place when u sit very much and you are amble to lower this. The risk of new health problems like a new infarct, but also other conditions such as diabetes mellitus reduce substantially! The largest health benefits can be achieved when your daily sedentary time is high at the moment. When you are able to reduce the daily sitting time, the chance is larger that you will live longer and healthier. Ask the patient how he thinks about that: [What does this mean to you for you daily life?]*

*When we look at the figure on the right side, you see the amount of continuous sitting hours per session from left to right [point out]. From below to the top, health is shown again. When you continuously sit more than 1 hour, you come in the risk zone as you can see. When you can limit continuous sitting bouts to maximal 1 hour, you improve your health. When you are able to stand up every half our, walk a bit, take the stairs, you come in the safe, green zone and you get the largest health benefits.*

D. More physical activity improves health!

Besides sitting less, you can improve your health by regular physical activity. It is advised to be physicialy active for at least 150 minutes a week, so for example 30 minutes of activity 5 times a week. These activities do of course also include for example walking and cycling! Especially when you are inactiv at the moment, you can improve your health by exercising regularly. Let’s have a look at the Figure below to see the effects of physicial activity on health.


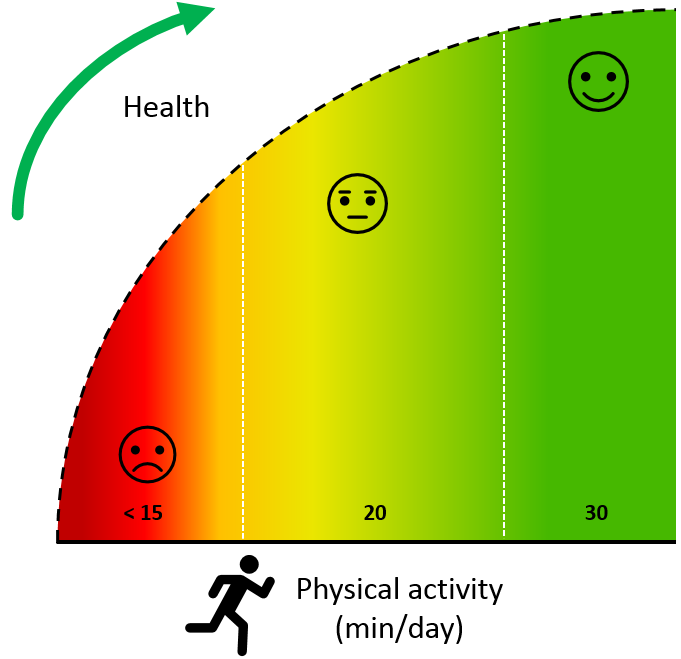


Explanation by the Figure S2-2D above:
*As you can see in the Figure, health is shown from the bottom to the top and the amount of physicial activity varying from 15 minutes/day to 30 minutes/day is shown from left to right. Especially when you have less physical activity at the moment, the health effect will be large when you increase the daily amount of physical activity to 20 or 30 minutes a day.*

Check is the patient understands all the new information correctly, correct possible misconceptions and enforce correct ideas. Let the patient activly process the information in a short discussion:

- *Recalling what we just discussed:*
  - *What do you think of this information?*
  - *What are ways to improve health?*

*Answers: 1) sitting less 2) break up long sitting bouts by standing up more regularly and 3) more physical activity. When the patients is able to combine these 3 points (sitting less AND breaking up sitting time more often AND more physicial activity), this can lead to maximal health benefits!*

- *What may be health benefits of sitting less/more physical activity on the short and long term?*

*Answer: the chance of a longer and healhier life increases, the risk of health problems reduces (for example: a new heart infarction, other conditions like diabetes mellitus).*

- *What are personal reasons for you to improve health?*

*Example answers: I can lose weight, I will feel better, I can bring my grandchildren to school by bike.*

It is important to get insight into personal reasons and possible concerns regardings sitting less to achieve health benefits. We would like to help you. Do you want to hear more about that?

1. Reasons and concerns regarding sitting less

AIM: Discuss the motivation to reduce sedentary time and go through advantages and disadvantages that are often named, added to reasons and concerns of the patient. Enforce identified advantages and (if inaccurate) correct identified disadvantages. New advantages are introduced.

CONTENT: In this step, the advantages of sitting less are discussed and possible concerns and doubts are addressed. Misconceptions are corrected.

1. Reasons to sit less

When we recall the mentioned advantages of sitting less, what is the most important reasons for you to sit less? [start a conversation based on the answer of the patient]

[if the patient has difficulties with mentioning a reason]: When you are okay with it, we can look a list of answers that other patients gave and you can mention if you recognize yourself in their points:

By reducing the daily sedentary time/break up sitting:

1. I reduce the risk of a new heart infarction in the future
2. I feel fitter
3. I have more energy
4. I can walk/cycle further
5. I reduce the burden of stiffness/joint problems
6. I stay mobile for a longer time
7. I reduce the risk of early mortality
8. I experience less stress
9. I can bring my grandchildren to school/sports
10. I care for a beneficial effect on my:
    1. Blood pressure
    2. Weight
    3. Cholesterol
    4. Blood sugar level
    5. Blood vessels
11. I feel myself better mentally
12. I stay able to take care of my partner
13. I can bet here for my partner / children / grandchildren in the future
14. I stay independent as long as possible
15. Other, namely …
16. Concerns regarding sitting less

What are personal possible disadvantages or concerns regarding sitting less? [start a conversation based on the answer of the patient]

[if the patient has difficulties with mentioning concerns]: When you are okay with it, we can look a list of answers that other patients gave and you can mention if you recognize yourself in their points:

I have concerns about sitting less or there are obstacles that needs to be overcome because:

1. I am concerned if my heart is able to manage
2. I don’t know how to combine it with other obligations in my daily life (like seated work for example)
3. I have a limited condition
4. I have less energy
5. I am easily tired
6. I don’t know how I can do it
7. I have other condition that limit me (like rheumatoid arthritis, joint degeneration, varix)
8. I cannot stand or move for a long term.
9. I am afraid that my daily life is interrupted by it (for example watching television sitting on the couch or chair)
10. I don’t experience direct health benefits of reducing my sedentary time
11. I don’t see the point of sitting less
12. I perform physical activity regularly and don’t believe that sitting less has additional benefits*
13. Other, namely …

*Add 12. When this concern is mentioned by the patient: exercise cannot fully compensate for the disadvantages of prolonged sitting on health. That means both are important for your health.

We have now discussed the advantages and possible obstacles surrounding sitting less that are applicalbe in you personal situation.

Do you have the feeling that the advantages to lower your sedentary time and perform more physical activity outweigh your concerns and disadvantages?

Shall we have a look how you can lower your sedentary time?

1. Goal setting daily sedentary time

AIM: Go through the different scenarios of daily sitting time and the set goal. The patient selects a scenario and motivates why this scenario suits him/her. Thereafter, the reason for choosing this scenario is discussed whereby language of change is provoked. Motivating reasons are enforced, while reasons why the goal may not be reached are further explored.

CONTENT: In this step, the daily sedentary time goal is set. Based on the selected scenario, the patient is asked why he/she did not raise the bar higher (disadvantages of sitting less are mentioned) and subsequently why the bar is not set lower (advantages of sitting less are mentioned and language of change is provoked).

1. Scenario selection + goal setting

You will receive the activity tracker (Activ8 sit tracker) for the upcoming 12 weeks. This tracker registrates not only how long you sit, stand or walk, but gives also a buzz signal in case an uninterrupted sitting bout of more than 30 minutes is detected to remind you to stand up. By using the Activ8 sit tracker, you will get a clear overview of your daily sedentary time.

Below, you can see 4 different graphs (**Figure 4A**). These are the 4 different examples of heart patients in which we measured sitting time using the Activ8 sit tracker.

You can see the different days of 1 month from left to right. The grey dots represent weekend days. The total sitting hours per day can be seen from the bottom to the top. The blue line shows the sitting goal that was set togheter with the patient. The orange bars [point out] represent the days that the total sedentary time was higher than the goal. The green bars [point out] represent the days that the total sedentary time was lower than the goal.

When we look at the 4 graphs, you can see a person that reaches the goal every day in graph A [point out]; all the bars are green. In graph B [point out], you see a person who does not reach the goal 1 or 2 days a week, but who sits less than the goal for most of the time; almost all the bars are green. In graph C [point out], you see a patient who sat more than the goal for most of the time, but reached the goal at some days; almost all the bars are orange. In graph D, the patient does not reach the goal all the days of the month; all bars are orange.

Set a sedentary behaviour goal for the upcoming weeks in consultation with the patient

[When you look back at the graph below the section *Sitting less and stand up more often improves health!*], what is your estimation regarding you total sedentary time a day and where do you want to set the goal?

I set my sitting goal at maximal … hours per day the upcoming week.

[When the patient has difficulties with estimating his/her daily sitting time / setting the first goal]: The average sitting time of a CVD patient is 10 hours a day [point out on the graphs below the heading *Sitting less and stand up more often improves health!*]. That is why we set the goal 1 hour per day lower. We will evaluate the goal during the next telephone consultation in 1 week. If needed, we will adjust the goal dependent on the measured sitting time.

Scenario selection

When you look at the Figure S2-4A below, how would you like to see your sedentary behaviour report displayed the upcoming month? Please choose between scenario A – B – C – D.

- [Reflection after the choice] > You do you chose for scenario X and not scenario X+1?
  (X=chosen scenario, X+1= 1 better than the chosen scenario, ask why not A when the patient choose for B for example. If scenario A is chosen; may it be possible to lower the goal to further reduce sitting time?)

[Get insight in the possible barriers for change and elaborate on the mentioned points]

- [Reflection after the choice] > What makes it possible that you chose for scenario X instead of for example scenario X-1? (X=chosen scenario, X-1= 1 worse than the chosen scenario, ask why not C when the patient choose for B for example)

[Reasons for change are mentioned and enforced]

**Figure S2-4A Different scenarios of daily sedentary time**


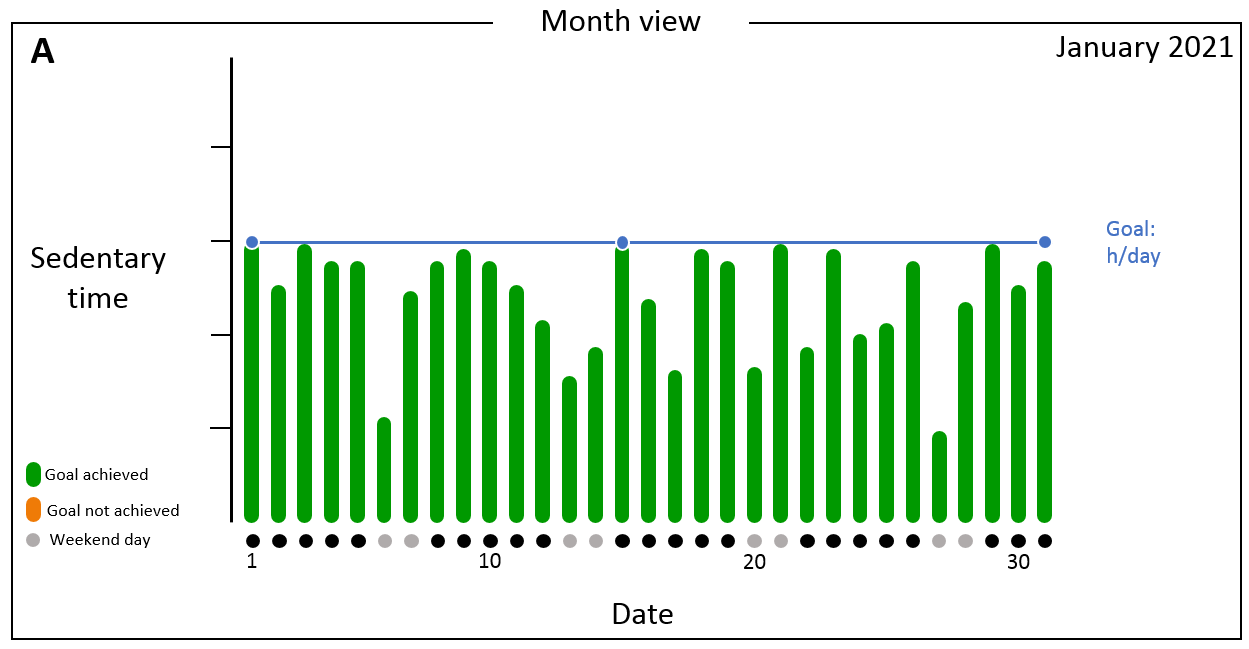

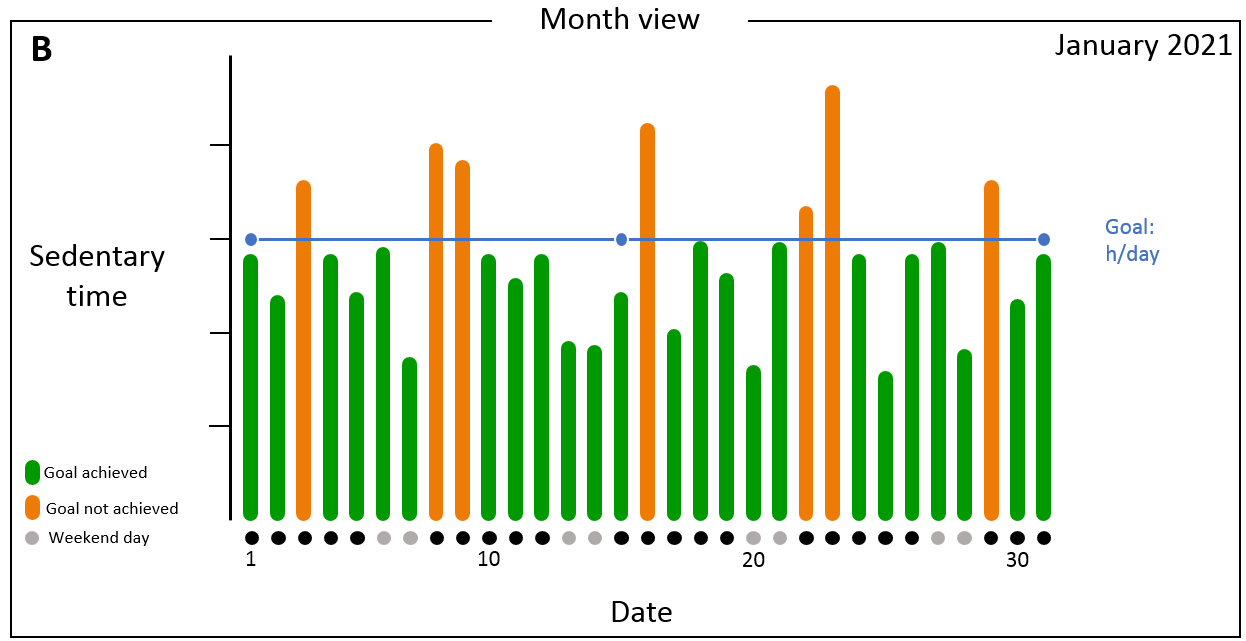

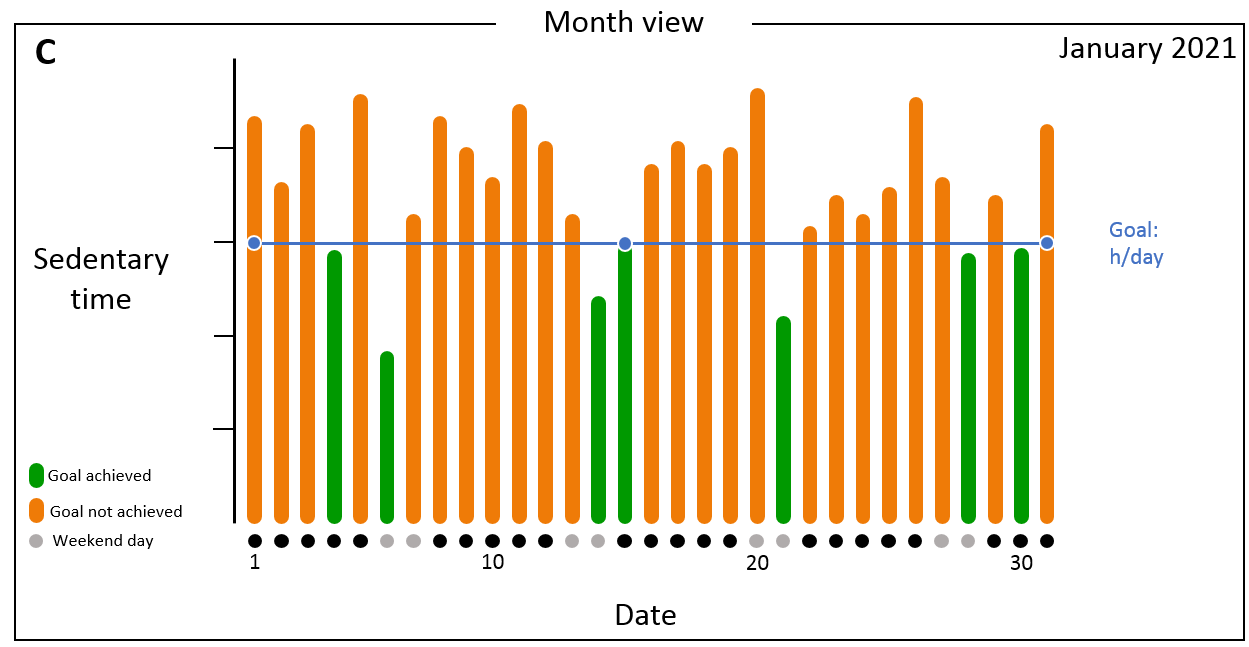

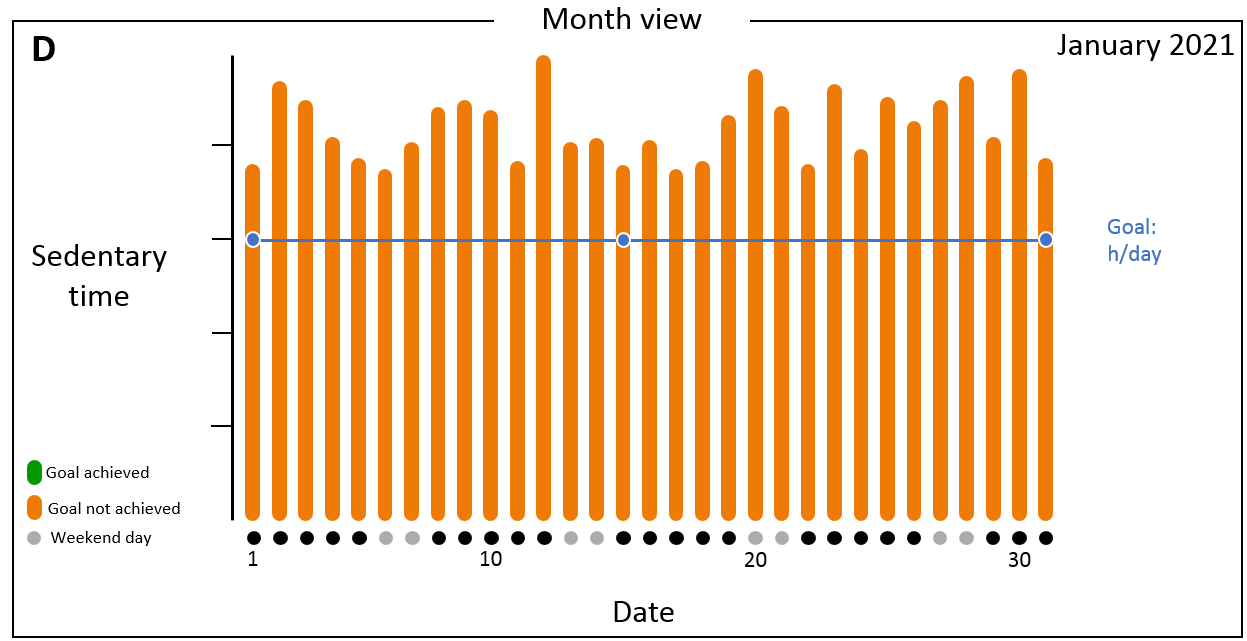


**Different scenarios of daily sedentary time.** Four different examples of sedentary behaviour monitoring reports derived from the activity tracker. On the X-axis, the days of 1 month are shown. The grey dots represent weekend days. On the Y-axis, the total sitting hours per day are shown, whereas the blue line represents the sitting goal that is set. In case the sedentary time was higher compared to the goal, the representing bar is orange, and in case the sedentary time was lower than the goal, the representing bar is green. In panel A, all the bars are green, representing a patient that reaches the goal every day. In panel B, almost all bars are green, meaning that this patient generally achieves the goal, except for 1 or 2 days per week. In panel C, almost all bars are orange, which indicates that sedentary time was higher than the goal most of the days during this month and only achieves the goal for a couple of days. In panel D, the patient is not able to reach the sitting goal all days of the month.

1. Define a plan to achieve goals, discuss difficult situations and possible solutions

AIM: Make a concrete plan to lower daily sitting time and to break up sitting every 30 minutes. We discuss specific, personal situations with the patient and anticipate on foreseen problems by thinking about possible solutions. This is done to improve the chance that the patient is able to reach the goal.

CONTENT: In this step, a concrete and specific plan is made to sit less and break up sitting more often. Possible problems are address and possible solutions are considered.

1. Possibilities, difficult situations and solutions to sit less

In the previous step, we set a first goal regarding maximal daily sitting time. It helps to make a plan how to reach that goal on forehand. The chance of success is larger with a clear plan.

Identify:

- What can you do to reach the goal and sit less?
- What do you need for that? [use the scheme below]

Possibilities to sit less in your daily life to reach the goal:

1. ………………………………………………………….………………………………………………………….

………………………………………………………….………………………………………………………….

1. ………………………………………………………….………………………………………………………….

………………………………………………………….………………………………………………………….

1. ………………………………………………………….………………………………………………………….

………………………………………………………….………………………………………………………….

By thinking about possible obstacles when executing the plan, we can discuss solutions to succeed in lowering sedentary time in those difficult situations.

Are there possible obstacles that may arise when lowering your sitting time on forehand? What may help you to reach your goal in those situations? [use the scheme below]

Possible obstacle: Solution:

1. …………………………………………………………. ………………………………………………………….

…………………………………………………………. ………………………………………………………….

1. …………………………………………………………. ………………………………………………………….

…………………………………………………………. ………………………………………………………….

1. …………………………………………………………. ………………………………………………………….

…………………………………………………………. ………………………………………………………….

[When the patient has difficulties with mentioning difficult situations to reach the sitting less goal him/herself]: When you are okay with it, we can have a look at a list with experiences of other patients. You can mention is the obstacle and corresponding solution might also apply to you or maybe you come to new insights yourself:

Other patients had difficulties with sitting less in the following situations. Do you recognize yourself in one of those situations? [When an obstacle is identified:] What would be a possible salutation for you in this situation? [When the patient has difficulties with mentioning possible solutions him/herself, discuss than the example solutions that are based on experiences of other patients. See below]:

- I find it difficult to lower sedentary time when watching television
  *Possible solutions:*
  - [solution of the patient] ……………………………………………………………
  - Watch television when standing during short programs such as the news
  - Pause the tv program / movie every 30 minutes to stretch the legs
  - Stand up, stretch the legs and/or walk during every commercial break
- I am not able to lower sedentary time with bad weather
  *Possible solutions:*
  - [solution of the patient] ……………………………………………………………
  - Search for physical activity possibilities inside:
    - Walk up and down the stairs
    - Take the stairs instead of the elevator
    - Go to the toilet on another floor
  - Do activities inside such as:
    - Cleaning
    - Cooking
    - Do home repairs
    - Exercise (home trainer, yoga, [other])
  - Take an umbrella and wear suitable outerwear
- It is difficult to lower my sedentary time during work, because I have a seated job.

*Possible solutions:*

- - [solution patient] ……………………………………………………………
  - Go for a walk during the coffee or lunchbreak (take coffee / tea in a thermos)
  - Do not let colleagues bring you coffee / tea, but get coffee / tea yourself
  - Place the bin behind the corner
  - Have a look at the possibilities of work that can be done while standing (standing desk, standing / walking meetings) and discuss it (when possible) with colleagues and managers.
- I forget to sit less sometimes

*Possible solutions:*

- - [Solution of the patient] ……………………………………………………………
  - Ask your partner / close friends / family to remember and encourage you to sit less
  - Couple it to a daily routine:
    - Before I brush my teeth, I walk through the house
    - When I come home, I go for a walk first
    - After supper, I go for a walk first
    - When I set my alarm for the next day, I think about moments to move during the next day
- When I travel by bus/train, it is difficult to sit less

*Possible solutions:*

- - [solution of the patient] ……………………………………………………………
  - Stand up (regularly) when traveling by bus / train
  - Walk a bit before going by train / bus / taxi (instead of sitting on a bench)
  - Get out of the bus one stop earlier and walk the last part to your destination
- I read the newspaper while sitting for 1,5 hours at the beginning of the day

*Possible solutions:*

- - [Solution of the patient] ……………………………………………………………
  - Read the newspaper while standing (use a table riser or kitchen counter for example)
  - Spread reading the newspaper over the day; for example, in the morning and evening half an hour.
- I feel tired / don’t feel like sitting less sometimes

*Possible solutions:*

- - [solution of the patient] ……………………………………………………………
  - Take small steps: for example, 3x 10 minutes of walking instead of 1x 30 minutes
  - Search for ways that suit you, for example standing activities when walking is not possible
  - Spread short physical activity moments over de day

Discuss if the solutions mentioned above may help the patient and if there are other solutions that suit the personal situation of the patient.

1. A plan to sit less

Make a concrete plan to lower sedentary time together with the patient. Include foreseen problems and possible corresponding solutions [when situation X occurs, I do Y].

I which situations are you able to break up sitting and lower the total sitting time? What are you going to do in these situations? [Make use of the scheme below]:

When X occurs: I perform Y:

1. …………………………………………………………. ………………………………………………………….

…………………………………………………………. ………………………………………………………….

1. …………………………………………………………. ………………………………………………………….

…………………………………………………………. ………………………………………………………….

1. …………………………………………………………. ………………………………………………………….

…………………………………………………………. ………………………………………………………….

[When the patient has difficulties with making a concrete plan]: when you are okay with it, we have a look at a list of examples how other patients have dealt with it. You can say if this plan may also work for you or maybe this leads to inspiration for new plans:

1. When the Activ8 sit tracker buzzes, I *stand up / walk up and down the stairs / walk to the garden / other* ………………………………………………………….
2. When I drive to *my work / children / brother / sister / the grocery store / the gym / other*………., I park my car a bit further so I can walk the last part
3. When I finish breakfast, I stand up and go *walking / cycling / go up and down the stairs / other………………………………………………………….*
4. When I am at work and sat for 30 minutes, I *stand up / walk / get some coffee / make tea / other ………………………………………………………….*
5. When I make a phone call, I *stand up / walk / other ………………………………………………………….* while calling
6. When I watch television, I *stand up during every commercial break / walk up and down the stairs / walk / take something to drink / go to the toilet on another floor (when possible) / other* …………………………………………………..
7. When I *get up / go to* bed, I think about the moments that I can go *for a walk / cycling / other………………………………………………………….* This way, I introduce a small physical activity moment every day

Now we have a clear plan, the chance is larger that you are able to reduce your sitting time in the future. In the next step, we set your goal in the online environment of the Activ8 sit tracker and discuss how you can reach that goal based on your plan. Shall we move on to the next part?

1. Evaluate predefined goals and determine confidence in achieving them

AIM: Set a (new) goal in the online environment of the Activ8 sit tracker. Thereafter, we discuss the confidence of the patient to reach the goal.

CONTENT: In this step, the maximal daily sitting time goal is set. Thereafter, we define the confidence of the patient to reach that goal using the defined plan. We discuss potential barriers with the patient and enforce reasons for reaching the goal.

Maximal sitting time – goal for the next time

We have discussed prolonged sitting as a risk factor for cardiovascular disease and we talked about reasons and potential obstacles when reducing sedentary time that apply to your personal situation.

You mentioned before that scenario [chosen scenario] suits you best. We have set your personal goal at a maximal sitting time of … hours per day. I will set this goal in the online environment of the Activ8 sit tracker. You can see in the app on your phone if you reached your sit goal [set the goal in the online environment of the Activ8 sit tracker]. Of course, we can adjust the goal during if needed during the telephone consultation next week.

I want to ask you to note in which moments you are able or unable to reach the goal the upcoming week. Note also what helped for you in these moments.

1. Confidence in the ability to accomplish the goal

How much confidence do you have to be able to reduce the daily sitting time to maximal [goal set] hours/day on a scale ranging from 1 to 10?

1……..2……..3……..4……..5……..6……..7……..8……..9…...10

- By high confidence (8 or higher):

Enforce/confirm (good to hear that you have a high confidence in succeeding!)

1. What makes it a 8/9/10?
2. Can you mention why you are able to lower the sitting time?

*[Reasons for change are mentioned and enforced]*

- By an average confidence (6 or 7)

Give a reflection (good to hear that you have confidence in succeeding!)

*Consider* to ask the next questions:

1. Can you mention what is needed to go from X to X+1? (X=current grade; x+1 = 1 grade **higher** than X) [optional]

[*Insight in possible barriers for change and elaborate futher on these points*]

1. You gave yourself a X and not X-2, that is really good! Can you mention why you are able to lower sedentary time? (X= current grade; X-2= 2 grades lower than X) [optional]

[*Reasons for change are mentioned and enforced*]

- By low confidence (lower than 6)

1. Can you mention what is needed to get a sufficient grade?

[*Insight into possible barriers for change and to eleborate on these points*]

1. Option 1: [a new barrier is mentioned]

Go again through the step where obstacles are identified and possible solutions are mentioned. After discussing a new plan; ask again to score the confidence.

Option 2: [the goal is too ambitious]

Set the goal a bit lower. It is okay to reduce sedentary behaviour with smaller steps.

Option 3: [patient makes a carefull estimation to protect him/herself for disapointment]

Accept and agree to see how it goes in practice. Emphasis that the goal can be adjusted when desired.

1. Monitoring sedentary behaviour – the Activ8 sit tracker

AIM: Short introduction of the Activ8 sit tracker and smartphone application to the starting patient

CONTENT: In this step, monitoring of the daily sitting time is discussed. The functionalities of the Activ8 sit tracker and smartphone application are explained. The supporting role of the activity tracker to continue the intervention to reduce sedentary behaviour at home is discussed. Telephone consultations throughout the intervention period are announced.

1. Introduction of the activity tracker

You are going to use the Activ8 sit tracker the upcoming 12 weeks. The Activ8 sit tracker is worn in the pocket of your jeans and registers if you are sitting, standing or walk. A buzz signal is given in case an uninterrupted sitting bout of 30 minutes is detected. This signal reminds you to stand, walk or move for at least 5 minutes. In situations where this is not possible, this function can temporally be paused (for example in a traffic jam, during a meeting, at a funeral or in the cinema).

The Activ8 sit tracker is connected wireless to your mobile phone. When connected, you can see your daily sitting and activity time on your smartphone. It is important to take the Activ8 sit tracker out of your pocket when you are going to sleep. You can charge the Activ8 sit tracker by connecting the tracker to a mobile adapter or to connect the tracker with a laptop/pc via the USB-connection. You will receive a manual so that you have all the information to use the Activ8 sit tracker at home. If needed, we are happy to assist you by telephone.

In the overview on your mobile phone, you can see your daily sitting time in the past week. You can also see if you succeeded in reaching your goal. Besides data over your daily sitting time, you can also see your average activity time. During the clinical visits, we will evaluate how it goes and you get personal advice to improve your sedentary behaviour. Potential problems are identified and we will search together for possible solutions. The personal goal can also be adjusted in consultation with you. We will contact you by phone between the consultations to coach and guide you. If indicated, the goal can be adjusted during the telephone consultations. That is why it is important to wear the Activ8 sit tracker on a daily basis and to charge the tracker regularly (i.e. at least once per week). When you wear clothes where it is impossible to wear the Activ8 sit tracker in the pocked, the tracker can be put in a small bag or sock and attached to the inside of your trousers / skirts / dress with a safety pin.

Is it clear how the Activ8 sit tracker can assist you to lower your sitting time?

1. Learning, problem solving and maintenance of behaviour change

AIM: Evaluate sedentary behaviour over the past weeks/months and make concrete plans for the upcoming period.

CONTENT: In the follow-up session, the achievements are discussed, possible problems and solutions are attended, goals may be adjusted and new plans are made for the upcoming period.

1. Evaluation of sedentary behaviour report

- How did it go the last weeks? When were you able to sit less and what were difficult situations? [enforce succes / overcoming difficult situations and discover reasons for less good experiences]
- How did the use of the activ8 sit tracker go? Where there days that you were not able to wear the activ8 sit tracker? How did you experience the buzz signal every 30 minutes?
- You set the goal at sitting maximal X hours/day and choose scenario X [show the graphs from *goal setting daily sedentary time*] during the last consultation. Do you have the feeling that you succeeded? Shall we have a look at your own results in the past weeks?
- What do you think?
  - Disappointing:
    - Show empathy and focus on positive points
    - See it as a learning experience and a chance to do it differently in the upcoming period to keep the confidence and motivation.
  - Goal is reached/better than expected:
    - Enforce the result and compliment the patient with his/her efforts

1. Learning, problem solving and maintenance of behaviour change

[When the goal is not reached]:

- Identify patterns for not reaching the goal in the past weeks. Differences between week and weekend days in reaching the goal? Specific days? Coincidence? [identify the cause]
- Did you experience obstacles when reaching your goal of sitting less in the past weeks? [fill in] Take potentially the often heard problems/solutions of other patient from the part *Define a plan to achieve goals, discuss situations and possible solutions*
- [When a possible problem is identified]: What might be future solutions for you to be able to lower sedentary time in the upcoming weeks?

Experienced obstacles: Effective solutions:

1. …………………………………………………………. ………………………………………………………….

…………………………………………………………. ………………………………………………………….

1. …………………………………………………………. ………………………………………………………….

…………………………………………………………. ………………………………………………………….

1. …………………………………………………………. ………………………………………………………….

…………………………………………………………. ………………………………………………………….

- Make a concrete plan and formulate specific actions based on the experienced problems and identified solutions [When situation X takes place, I perform Y].
- How much **confidence** do you have in succeeding to lower you daily sitting time to maximal [goal set] with the new plan on a scale ranging from 1 to 10?

1……..2……..3……..4……..5……..6……..7……..8……..9…...10

- When the patients scores high (8 or higher): enforce and confirm this
- When the patient scores lower (7 or lower): Ask why the confidence is not higher. What is needed to increase the grade?
  - Option 1: The goal is too ambitious
    - Set the goal a bit lower. It is okay to lower the sedentary time in steps
  - Option 2: New barriers are identified.
    - Discuss the concrete plan based on the new barrier and add new solutions
  - Option 3: patient makes a carefull estimation and does not want to be disappointed again
    - No specific problems to discuss
- Set the new goal in the online activ8 sit tracker environment.

[If the goal is reached]:

- Set a new goal: select again a maximal sit goal and according scenario based on the graphs in the section *Goal setting daily sedentary time*.
- How are you going to reach the new goal?
  - Did you work a little harder than normally to reach the goal?
  - Are you able to carry on with this behaviour?
  - What do you need for that?
  - How does this new behaviour become daily routine?
  - Are there factors that can break this new good routine and how can you deal with them? Use potentially the often named problems/solutions of other patients below the section *Define a plan to achieve goals, discuss situations and possible solutions*

Possible future obstacles: Possible solutions:

1. …………………………………………………………. ………………………………………………………….

…………………………………………………………. ………………………………………………………….

1. …………………………………………………………. ………………………………………………………….

…………………………………………………………. ………………………………………………………….

1. …………………………………………………………. ………………………………………………………….

…………………………………………………………. ………………………………………………………….

- Make a new concrete plan focused on maintaining this new good behaviour
- Agree that the patient continuous using the activ8 sit tracker until their current behaviour is a routine

Closing

- Ask the patient to note the reasons for not reaching the sitting goal, when the patient experiences difficulties, and which solutions are found by the patient.
- Sum up the conversation and ask the patient if everything is clear [activ8 sit tracker is set again, concrete plan is made, goals and appointments are formulated.
- Finish by informing the patient that the telephone consultation will take place once every two weeks and ask the patient to contact the research theme when he/she experience problems earlier.
- An ActivPAL activity tracker is placed on the upper leg during the last SIT LESS intervention consultation. After 1 week, the ActivPAL is removed from the upper leg by the patient and together with the Activ8 sit tracker, the ActivPAL is send back by registered mail parcel.
